# Anti-Alzheimers molecular mechanism of icariin: insights from gut microbiota, metabolomics, and network pharmacology

**DOI:** 10.1186/s12967-023-04137-z

**Published:** 2023-04-24

**Authors:** Yuqing Liu, Hongli Li, Xiaowei Wang, Jianhua Huang, Di Zhao, Yejun Tan, Zheyu Zhang, Zhen Zhang, Lemei Zhu, Beibei Wu, Zhibao Chen, Weijun Peng

**Affiliations:** 1 Department of Integrated Traditional Chinese & Western Medicine, The Second Xiangya Hospital, Central South University, No.139 Middle Renmin Road, Changsha, Hunan 410011 People’s Republic of China; 2 National Clinical Research Center for Metabolic Diseases, The Second Xiangya Hospital, Central South University, Changsha, 410011 China; 3 Department of Pathology, The Second Xiangya Hospital, Central South University, Changsha, 410011 Hunan Province China; 4 Hunan Academy of Chinese Medicine, Changsha, 410013 People’s Republic of China; 5 School of Mathematics, University of Minnesota Twin Cities, Minneapolis, MN 55455 USA; 6 YangSheng College of Traditional Chinese Medicine, Guizhou University of Traditional Chinese Medicine, Guiyang, 550025 Guizhou China; 7 Academician Workstation, Changsha Medical University, Changsha, 410219 China

**Keywords:** Icariin (ICA), Alzheimer’s disease (AD), APP/PS1 mice, *Akkermansia*, *Alistipe*, Sphingolipid metabolism, Network pharmacology (NP)

## Abstract

**Background:**

Icariin (ICA), an active ingredient extracted from *Epimedium* species, has shown promising results in the treatment of Alzheimer's disease (AD), although its potential therapeutic mechanism remains largely unknown. This study aimed to investigate the therapeutic effects and the underlying mechanisms of ICA on AD by an integrated analysis of gut microbiota, metabolomics, and network pharmacology (NP).

**Methods:**

The cognitive impairment of mice was measured using the Morris Water Maze test and the pathological changes were assessed using hematoxylin and eosin staining. 16S rRNA sequencing and multi-metabolomics were performed to analyze the alterations in the gut microbiota and fecal/serum metabolism. Meanwhile, NP was used to determine the putative molecular regulation mechanism of ICA in AD treatment.

**Results:**

Our results revealed that ICA intervention significantly improved cognitive dysfunction in APP/PS1 mice and typical AD pathologies in the hippocampus of the APP/PS1 mice. Moreover, the gut microbiota analysis showed that ICA administration reversed AD-induced gut microbiota dysbiosis in APP/PS1 mice by elevating the abundance of *Akkermansia* and reducing the abundance of *Alistipe*. Furthermore, the metabolomic analysis revealed that ICA reversed the AD-induced metabolic disorder via regulating the glycerophospholipid and sphingolipid metabolism, and correlation analysis revealed that glycerophospholipid and sphingolipid were closely related to *Alistipe* and *Akkermansia*. Moreover, NP indicated that ICA might regulate the sphingolipid signaling pathway via the PRKCA/TNF/TP53/AKT1/RELA/NFKB1 axis for the treatment of AD.

**Conclusion:**

These findings indicated that ICA may serve as a promising therapeutic approach for AD and that the ICA-mediated protective effects were associated with the amelioration of microbiota disturbance and metabolic disorder.

**Graphical Abstract:**

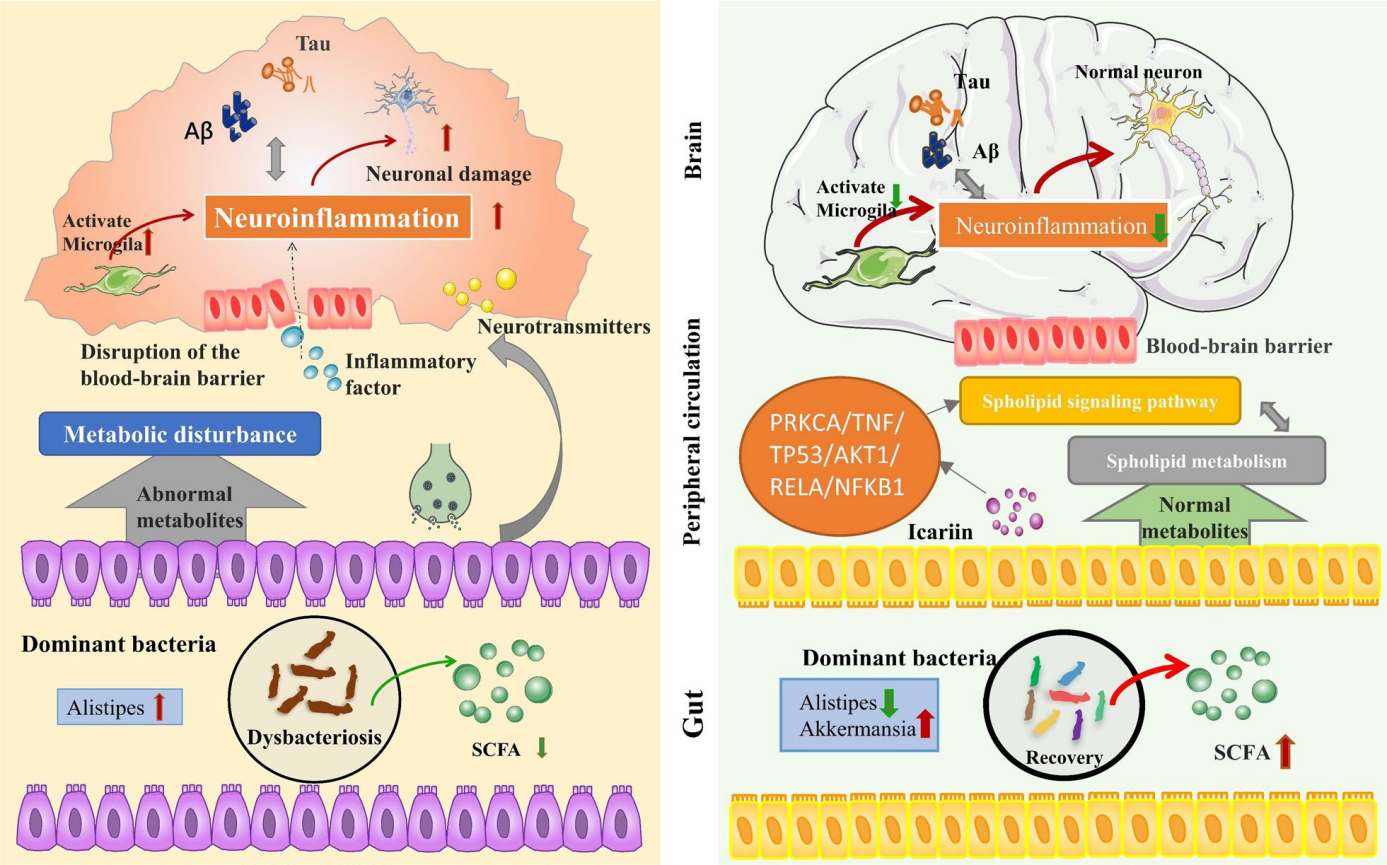

**Supplementary Information:**

The online version contains supplementary material available at 10.1186/s12967-023-04137-z.

## Background

Alzheimer's disease (AD) is a progressive neurological condition affecting over 35 million people worldwide. It is clinically characterized by cognitive impairment and memory deterioration, which occur in nearly 70% of all AD cases. Furthermore, AD is becoming a major global public health concern owing to its direct and indirect costs and the increasing aging population worldwide [1]. However, despite the considerable progress in AD research in the past decade, there are no effective treatments available for AD.

Icariin (ICA), an active component extracted from *Epimedium* species, has broad biological application prospects [2]. Numerous studies have shown that ICA and its metabolites can improve neuronal survival and function by reducing the production of extracellular amyloid beta peptide (Aβ) [3] and intracellular neurofibrillary tangles (NFTs) and inhibiting phosphodiesterase-5 activity [4]. Furthermore, ICA can inhibit the expression of proinflammatory factors, such as tumor necrosis factor (TNF)-α [5] and interleukin-1β [6]. Additionally, it can prevent H2O2-induced neurotoxicity by decreasing reactive oxygen species production by upregulating silent mating type information regulation 2 homolog- 1 (SIRT1) [7]. Furthermore, several studies indicate that ICA can cause alterations in the abundance, proportion, and function of gut microbiota in AD and age-related maladies [8, 9]. However, the underlying mechanism through which ICA mediates its anti-AD effects is poorly understood, thus impeding the development of ICA-based treatments for AD. Therefore, further investigations are warranted to explore the underlying anti-AD molecular mechanisms of ICA.

Multi-omics and multi-dimensional analyses are used to improve our understanding of disease development and drug therapeutic mechanisms. 16S ribosomal RNA (16S rRNA) gene sequencing is used for bacterial analysis, while metabolomics is used to track dynamic changes in metabolites (< 1000 molecular weight) [10], and can evaluate the metabolic effects of food [11] using high-throughput instruments. Lastly, network pharmacology (NP) is used to systematically explore bioactive substances [12]. Together, gut microbiota analysis, metabolomics, and NP we can be used to conduct in-depth research on the anti-AD effects of ICA.

In this study, the Morris Water Maze (MWM) test and hematoxylin–eosin (HE) staining were used to assess the impact of ICA on cognitive performance and histopathological changes in APP/PS1 mice. Additionally, 16 s rRNA analysis was used to examine the changes in the gut microbiota of APP/PS1 mice, and liquid chromatography–mass spectrometry (LC–MS)-based untargeted metabolomics was used to determine the regulatory effects of ICA on the metabolic profiles of the serum and fecal samples of APP/PS1 mice. Lastly, NP was used to assess the current scientific understanding of ICA in AD treatment. This study intends to shed light on the intricacies of AD biology and discover regulatory networks for the ICA-mediated treatment of AD.

## Methods

### Preparation of herbal extracts

ICA powder (≥ 98% purity) was purchased from (Nanjing Spring & Autumn Biological Engineering Co., Ltd., CH33H40015)). ICA was dissolved in 2% dimethyl sulfoxide (DMSO) and 10% polyethylene glycol 400.

### Animal modeling, grouping, and intervention

Animal studies were conducted according to the Guide for the Care and Use of Laboratory Animals by the National Institute of Health (USA) and were approved by the Animal Ethics Committee of the Second Xiangya Hospital, Central South University (China) (2020038). All the procedures were conducted under pentobarbital sodium anesthesia, ensuring minimal discomfort to the animals. C57BL6/J and APP/PS1 mice (6-month-old, 250–300 g) were obtained from Spife Biotechnology Co., Ltd. (Beijing, China). The mice were kept in a regulated habitat (50 ± 10% relative humidity, 12/12 h light/dark cycle, 22 ± 2 °C) with free access to standard food and water. APP/PS1 mice were randomly allocated into the model group (n = 10) and ICA group (n = 12), whereas C57BL6/J mice were assigned to the control group (n = 10). The ICA group was administered ICA at 100 g/(kg*d) for 100 d, whilst the other two groups received the same quantity of sterile saline solution.

### MWM test

The MWM test consists of spatial acquisition and spatial probe tests, which are used to assess spatial learning and memory abilities [12]. The spatial acquisition test was carried out by a 5-day memory acquisition experiment during days 94–98 to assess the spatial learning abilities of the mice. While the spatial probe experiment was carried out on day 99 to test the spatial memory retention abilities of the mice.

### HE staining

Following the MWM test, the mice were anesthetized and transcardially perfused with 300 mL of stroke-physiological saline solution to flush out their blood through the abdominal aorta. The blood samples were then stored at − 80 °C until further analysis. Thereafter, their brain tissues were collected, treated with a 4% paraformaldehyde solution, embedded in paraffin, and sectioned into 4–5 µm thick slices [13]. The sections were then HE stained and sealed with neutral gum. Finally, the sections were observed and photographed under an optical microscope.

### 16S rRNA gene sequencing analysis

#### Sample collection

A minimum of 5 fecal pellets were retrieved from the rectum of each mouse (100-d-old), placed in a sterile conical tube, and stored at − 80 °C until further analysis.

#### Data acquisition and processing

16S rRNA gene sequencing of the fecal samples was carried out as reported previously [14]. Briefly, the samples were subjected to polymerase chain reaction (PCR) amplification, after which the amplicons were purified and subjected to paired-end sequencing and the raw data was evaluated. Information on sequencing analysis is provided in the Supplementary Materials and Methods, and the data has been deposited in the National Center for Biotechnology Information Sequence Read Archive (BioProject ID: PRJNA918352).

### LC–MS

#### Sample preparation

The detailed protocol for fecal and blood sample preparation for LC-MS analysis is provided in the Supplementary Materials and Methods.

#### Data acquisition and processing

The UltiMate 3000 Ultra High-performance Liquid Chromatograph (Thermo Fisher, USA) and Thermo Q-Exactive Orbitrap Mass Spectrometer were used for ultrapure LC-MS/MS analysis (Thermo Fisher). Chromatographic separations were performed on Waters ACQUITY UPLC HSS T3 (100 mm × 2.1 mm × 1.8 μm). Thereafter, all the raw data were fed into Progenesis QI (Waters, Milford, MA, USA) and SIMCA-P14.0 (Umetrics AB, Umea, Vasterbotten, Sweden), software for further analysis [15]. The partial least-squares discriminant analysis (PLS-DA) and orthogonal partial least-squares discriminant analysis (OPLS-DA) supervised pattern recognition methods were applied to identify the overall metabolic differences among the three groups and the variable importance in the projection (VIP) was used to identify characteristic metabolites in the three groups. Distinct metabolites were analyzed based on VIP > 1.0 and P < 0.05. Further information is provided in the Supplemental Materials and Methods.

### Gut microbiota–metabolite correlation analysis

Firstly, we identified the differential metabolites in the control, model, and ICA groups, which are engaged in important metabolic pathways, via correlation analysis. Thereafter, we identified the top 45 most abundant species in the intestinal flora in all three groups. Lastly, we used Spearman correlation analysis and heatmaps to assess the correlation between gut microbiota and metabolite content.

### NP

The drug targets were predicted by PubChem (https://pubchem.ncbi.nlm.nih.gov/) [16], Pharm Mapper (http://www.lilab-ecust.cn/pharmmapper/), and Swiss Target Prediction (http://www.swisstargetprediction.ch/) databases, and all the targets were annotated as their official symbols using the UniProt Knowledgebase search function (http://www.uniprot.org/) in the Protein Database (UniProt). Thereafter, the GeneCards (https://www.genecards.org/) and Online Mendelian Inheritance in Man (OMIM) (https://omim.org/) [17] databases were used to obtain the AD-related genes. Among these, the promising therapeutic targets were submitted to the Search Tool for the Retrieval of Interacting Genes/Proteins (STRING) database (https://string-db.org/) for protein–protein interaction (PPI) network formation using Cytoscape 3.8.0. Thereafter, Gene Ontology (GO) enrichment and Kyoto Encyclopedia of Genes and Genomes (KEGG) pathway enrichment analyses were conducted to further investigate the biological functions of the targets and their associated pathways. Lastly, the hub genes associated with important pathways were selected for subsequent molecular docking analysis by PyMOL 2.4.0 and Autodock software. Further information on the analytic processes is provided in the Supplementary Materials and Methods.

### Statistical analysis

Data were presented as mean ± standard deviation (SD). The SPSS 20.0 software (IBM, Armonk, NY, USA) was used to conduct statistical analyses. The escape latency was analyzed by separate repeated measures of two-way analysis of variance (ANOVA). Other data were evaluated by one-way ANOVA. P < 0.05 was considered statistically significant.

## Results

### ICA alleviated cognitive impairment and histopathological changes in APP/PS1 mice

**Fig. 1:**
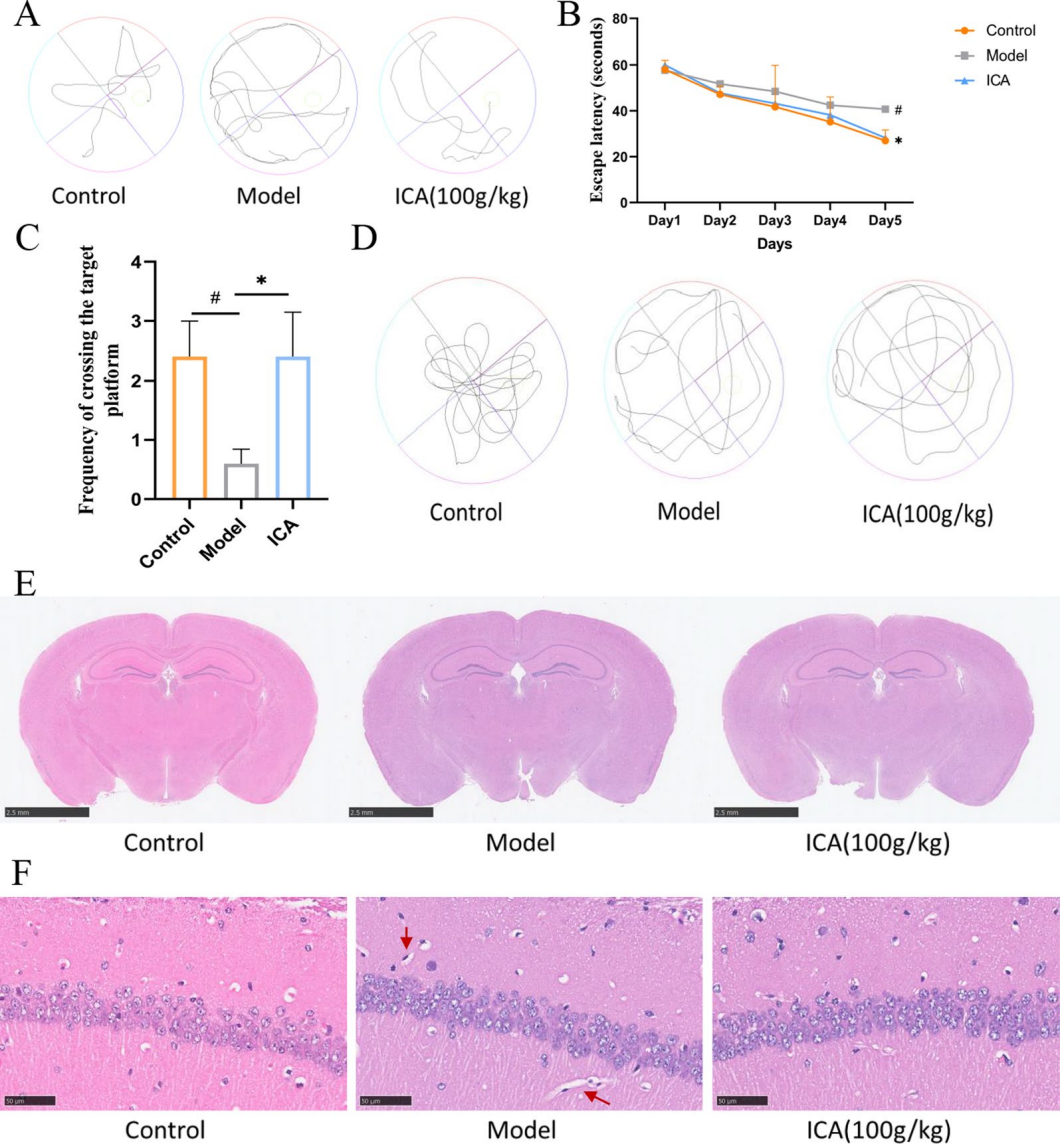
MWM test and HE staining of the control, model, and ICA groups. **A**, **B** Representative photos of the swim pathways **A** and escape latency **B** to locate the concealed platform during days 1–5. **C**, **D** The number of platform crossings **C** and the representative photos of target platform crossing frequency **D** on day 6, after removing the platform. Data are expressed as mean ± SEM (n = 6/group). *P < 0.05 and **P < 0.01 vs. the control group and #*P* < 0.05 vs. the model group. **E**, **F** HE-stained brain sections from each group. The red arrows indicate distorted nerve cells. MWM test, Morris Water Maze test, ICA, icariin; HE, hematoxylin and eosin

The MWM test was used to detect and evaluate the spatial learning and memory capacities of APP/PS1 mice to examine the effects of ICA on their cognitive deficits. The escape latency data of navigation trials decreased significantly with daily practice. On day 5, the spatial probe test was administered and the time spent in the target quadrant and their frequency of crossing the target quadrant was recorded. The results of the MWM test demonstrated that the APP/PS1 mice had a regular and discrete swimming trajectory, a diminished number of platform crossings, and a longer escape latency compared to the control group (*p* = 0.0148, Fig. 1B, D). The ICA-treated APP/PS1 mice showed an intricate and disordered swimming trajectory, elevated number of platform crossings (*p* = 0.0263, Fig. 1C, D), and significantly reduced escape latency compared to the vehicle-treated APP/PS1 mice (*p* = 0.0173, Fig. 1B). These findings indicate that ICA therapy might improve the spatial learning and memory deficits observed in APP/PS1 mice.

Additionally, HE staining was used to demonstrate the impact of ICA on the histopathological changes of APP/PS1 mice. Hippocampus, which is critical for learning and memory, is particularly susceptible to injury in the early stages of AD [18]. As seen in Fig. 1E, F the nerve cells in the hippocampus of the control group were neatly organized and spherical, with proper cell membranes and nuclei and no evident swelling or necrosis. In contrast, the nerve cells in the hippocampus of the model group were significantly disorganized, distorted, uneven in size and structure, and reduced in quantity. However, ICA treatment preserved the integrity of nerve cells and reversed nerve cell necrosis in the APP/PS1 mice. Altogether, these findings suggest that ICA therapy might alleviate learning and memory deficits as well as histological alterations in the APP/PS1 mice.

### ICA altered the composition of gut microbiota in APP/PS1 mice

**Fig. 2:**
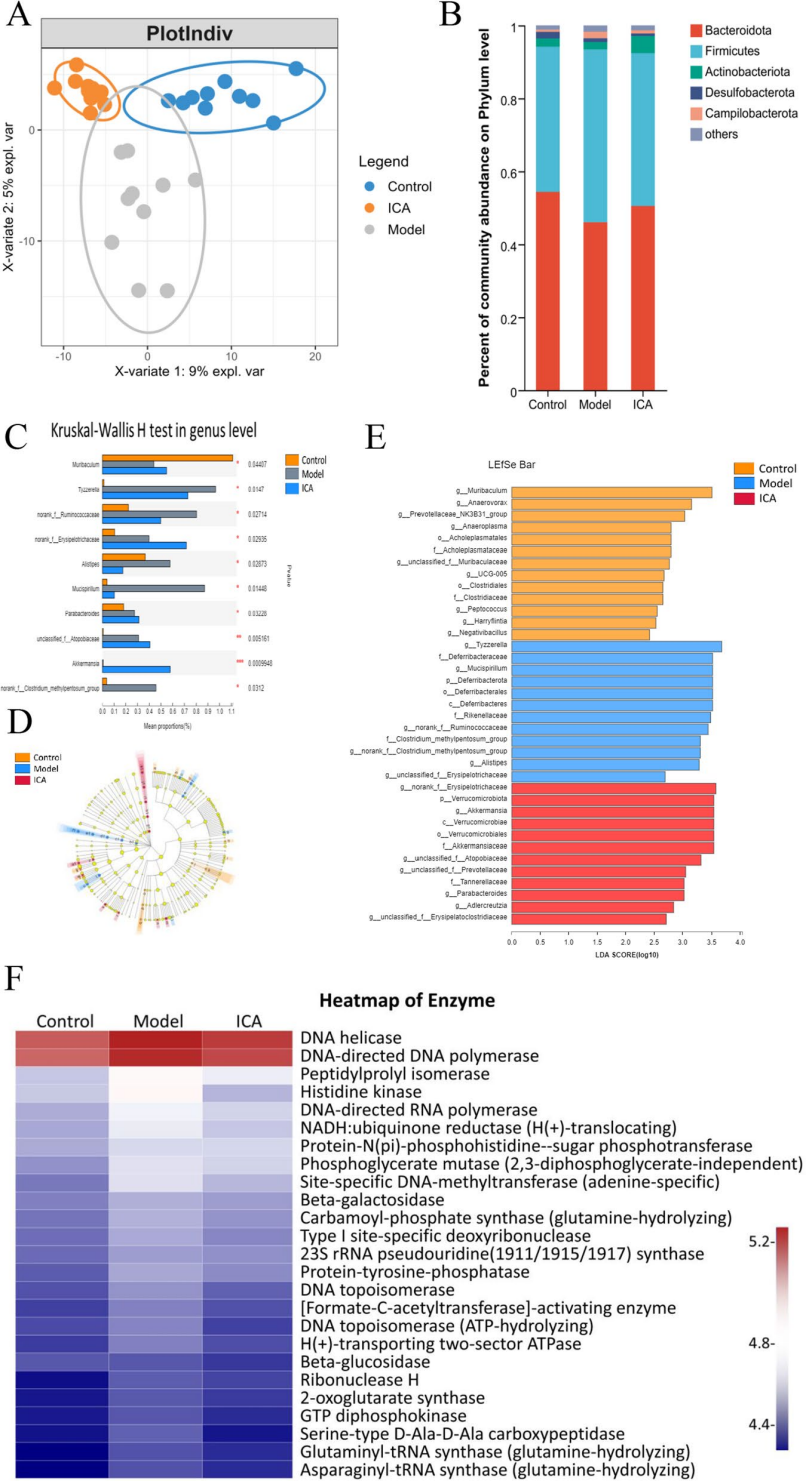
Analysis of the gut microbiota of the control, model, and ICA groups. **A** PLS-DA analyses of the three groups. **B** Percent of community abundance at the phylum level. **C** Bar plot of the Kruskal–Wallis test score of different species at the genus level. The Y-axis indicates the species name at the taxonomic level, X-axis indicates the average relative abundance in distinct groups of species, and the columns of varying hues represent the different groups. [The rightmost is the P value, * means *P* > 0.01 &lt; *P* ≤ 0.05, ** means *P* > 0.001 &lt; *P* ≤ 0.01, *** means *P* ≤ 0.001.] Cladogram of the phylogenetic distribution. The diameter of each circle is proportionate to the abundance of each taxon. **E** Histogram of the LDA scores of differentially abundant taxa between the control and model groups. **F** Heatmap of the functional analysis of enzymes. ICA, icariin; PLS-DA, partial least squares discrimination analysis; LEfSe, Linear discriminant analysis (LDA) effect size

To determine whether the gut microbiota is associated with the anti-AD effects of ICA in APP/PS1 mice, we conducted 16S rRNA gene sequencing of 32 fecal samples from the control (n = 10), model (n = 10), and ICA (n = 12) groups. The Ace and Chao indexes revealed that ICA treatment could restore the abundance of intestinal flora in AD mice (Additional file 1: Figure S1C, D), while the Shannon and Simpson indexes revealed that ICA treatment had no significant effect on the species richness in AD mice (Additional file 1: Figure S1A, B). Meanwhile, PLS-DA demonstrated the presence of distinct species in the three groups (Fig. 2A). To further evaluate the general makeup of the gut microbiota across the three groups, we examined the degree of taxonomic similarity between the bacteria. The results revealed that *Bacteroidetes* (46–54%) and *Firmicutes* (40–47%) were the leading phyla across the three groups (Fig. 2B). However, the *Firmicutes/Bacteroidetes* (F/B) ratio in the model group (1.02) was upregulated compared to the control group (0.74) and downregulated compared to the ICA group (0.86). The presence of certain lipid metabolism-associated bacterial species, such as *Akkermansia, Parabacteroides,* and *Alistipes* is regarded as a health concern. *Akkermansia* is associated with blood lipid and blood glucose metabolism [19], while *Alistipes* is associated with a higher-fat diet [20]. In this study, we found that the abundance of *Akkermansia* increased (*p* = 0.0009948, (Fig. 2C), while that of *Alistipes* decreased (*p* = 0.02873) after ICA intervention. Meanwhile, the abundance of inflammation-associated bacteria, *Mucispirillum *[21] decreased after ICA treatment (*p* = 0.04407). The linear discriminant analysis (LDA) effect size (LEfSe) was used to identify the key bacterial taxa in each group and all the validated sequences were analyzed (Fig. 2E). The ICA-treated mice exhibited an increased abundance of *Akkermansia*, while the model mice exhibited an increased abundance of *Mucispirillum* (Fig. 2D). The KEGG Pathway Database 16S rRNA data were predicted using PICRUSt (Fig. 2F) and the results revealed that the enhanced pathways in the ICA group were primarily associated with metabolism, especially glutamine, amino acid, and energy metabolism, with certain differences among the groups. Overall, these findings suggest that the ICA mediates its anti-AD effect via the microbiome–metabolism–brain axis.

### ICA reversed fecal metabolomics disorder in APP/PS1 mice

**Fig. 3:**
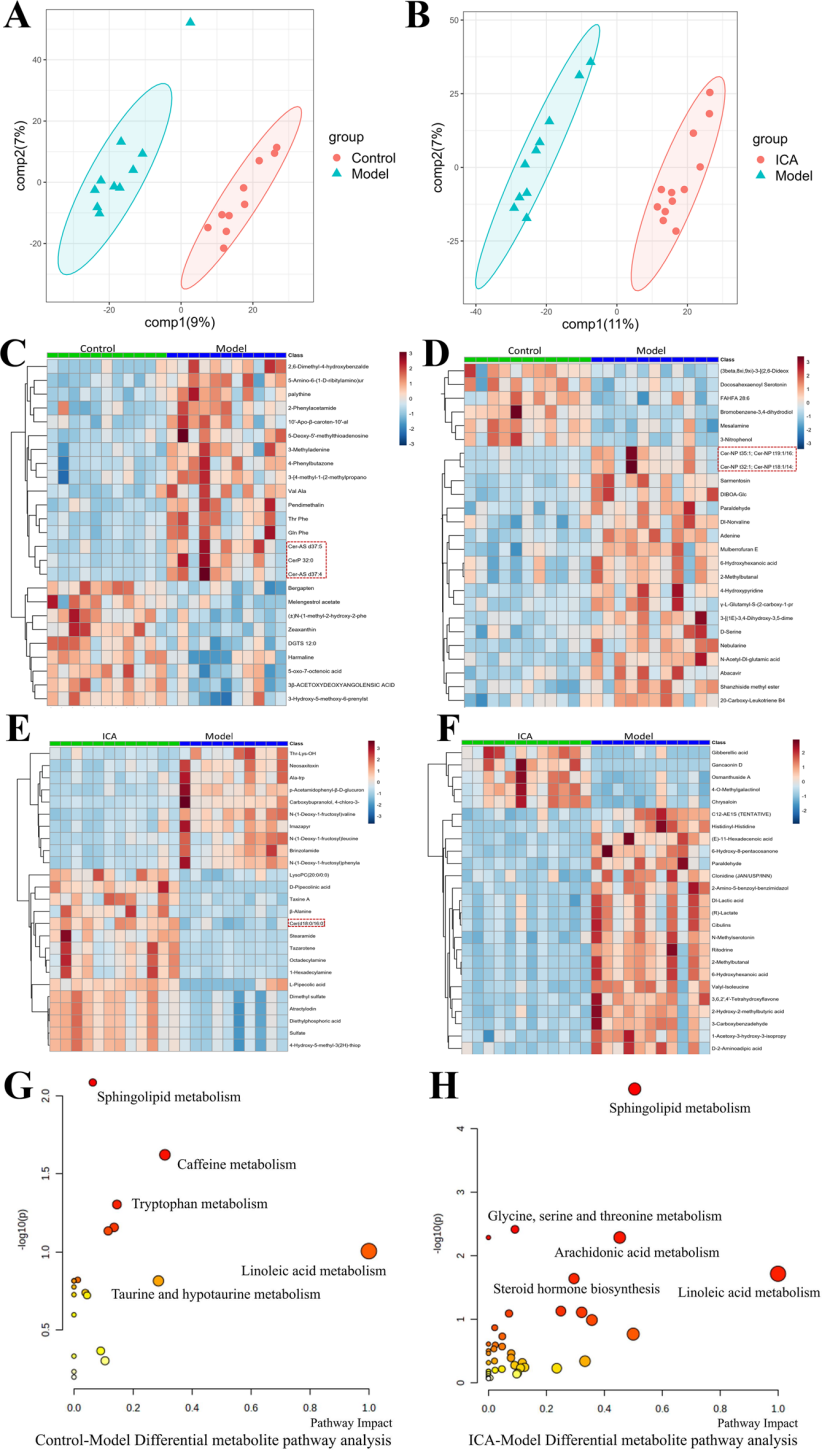
Fecal metabolomics of the control, model, and ICA groups. **A**, **B** PLS-DA analyses between the model **A** and ICA **B** groups. **C**, **D** Heatmaps of the differential metabolites in the control **C** and model **D** groups in the ESI + and ESI- modes. **E**, **F** Heatmaps of the differential metabolites in the ICA **E** and model **F** groups in the ESI + and ESI- modes. **G**, **H** Analysis of the pathways associated with the differential metabolites in the model **G** and ICA **H** groups. ICA, icariin; PLS-DA, partial least squares discrimination analysis; ESI, electrospray ionization

We further conducted fecal metabolomics on the control, model, and ICA groups to explore the anti-AD effects of ICA. PLS-DA and OPLS-DA (Additional file 1: Figure S2A, B) analyses identified the overall metabolic differences between the three groups and found obvious metabolic disorders in the model mice, which were partially reversed in the ICA-treated mice (Fig. 3A, B). Using VIP, we found a total of 380 differential metabolites (126 upregulated and 254 downregulated) between the control and model groups and 687 differential metabolites (265 upregulated and 422 downregulated) between the ICA and model groups. Among these, lipids and lipid-like molecules (17.5%) and fatty acyls (17.5%) were the two most prominent metabolites in all the groups (Additional file 1: Figure S2C). As seen in Fig. 3C–F, the abundances of the majority of fecal metabolites in the model group were considerably different from those in the control group and ICA treatment had a measurable influence on the fecal metabolome. Interestingly, sphingolipids showed significant differences among the three groups. Therefore, we examined the taxonomic makeup of the sphingolipid metabolites. The model group exhibited higher levels of sphingolipids, such as Cer-AS d37:5, Cer-NP t35:1, Cer-NP t19:1/16:0, and CerP 32:0, while the ICA group showed reduced levels of Cer-NDS d32:0, Cer 36:2, Cer-BS d35:1, and Cer-AP t42:0 (Additional file 1: Figure S2D). Furthermore, the pathway analysis showed that both AD and ICA contributed to changes in sphingolipid metabolism (*p* = 0.0081761). Altogether, these results demonstrate that ICA mediates its anti-AD function by reversing the effects of AD on the sphingolipid metabolism in the gut.

### ICA reversed serum metabolome disorder in APP/PS1 mice

**Fig. 4:**
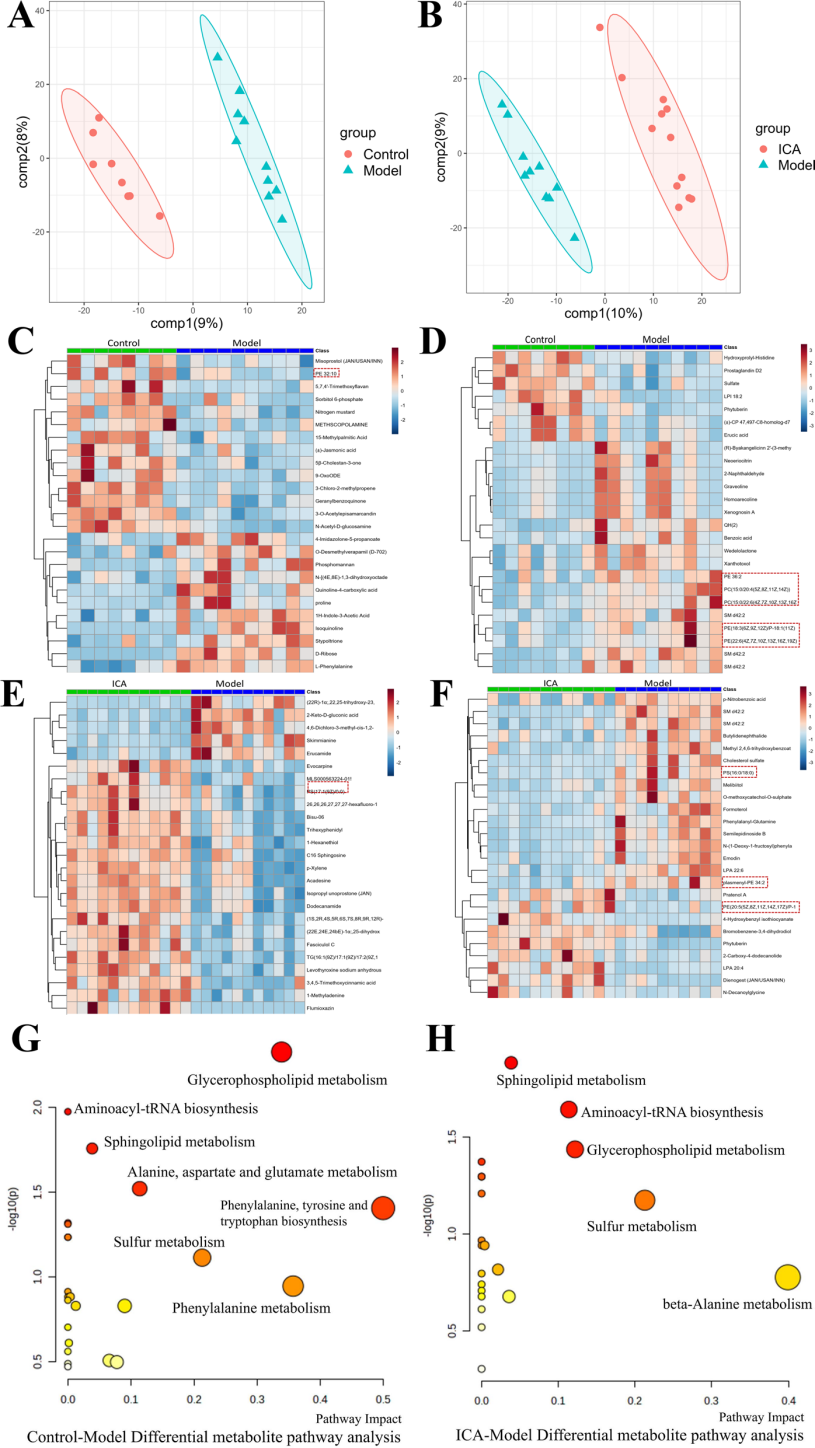
Serum metabolomics of the control, model, and ICA groups. **A**, **B** OPLS-DA analyses between the control **A** and model **B** groups. **C**, **D** Heatmaps of the differential metabolites between the control **C** and model **D** groups in the ESI + and ESI- modes. **E**, **F** Heatmaps of the differential metabolites between the ICA **E** and model **F** groups in the ESI + and ESI- modes. **G**, **H** Analysis of the pathways associated with the differential metabolites in the model **G** and ICA **H** groups. ICA, icariin; OPLS-DA, orthogonal partial least squares discrimination analysis; ESI, electrospray ionization

Our findings suggested that ICA affects the metabolic profile of the gut microbiota in APP/PS1 mice. We further conducted UPLC-MS/MS-based untargeted serum metabolomics to investigate the effect of ICA on the serum metabolites of the APP/PS1 mice. PLS-DA (Fig. 4A, B) and OPLS-DA analyses (Additional file 1: Figure S3A, B) showed that the model mice had obvious metabolic disorders, which were partially restored by ICA treatment. Using VIP, we found a total of 99 differential metabolites (45 upregulated and 54 downregulated) between the control and model groups and 111 differential metabolites (40 upregulated and 61 downregulated) between the ICA and model groups. Among these, lipids and lipid-like molecules (22.6%) were the most prominent metabolites in the ICA and model groups (Additional file 1: Figure S3C). ICA administration had a significant influence on the relative abundances of serum metabolites, particularly glycerophospholipids (Fig. 4C–F). Therefore, we explored the taxonomic distribution of glycerophospholipids and found that the levels of PE 40:9e, PE (18:1/22:6), and PI (16:0/20:4(5Z,8Z,11Z,14Z)) were significantly higher in the model group than in the control group, while those of PC (3:0/3:0), lysoPC 18:4, and PS (16:0/18:0) were downregulated in the ICA group compared to the model group. However, PE (20:5(5Z,8Z,11Z,14Z,17Z)/P-18:1(11Z)) and PC (18:2(15E,17E)/18:2(15E,17E)) [U] were also upregulated in the ICA group compared to the model group (Additional file 1: Figure S3D). Metabolic pathway analysis indicated the ICA may be associated with the regulation of glycerophospholipid metabolism (*p* = 0.0046882 in the control group vs. the model group and *p* = 0.036736 in the ICA group vs. the model group) and sphingolipid metabolism (*p* = 0.017438 in the control group vs. the model group and *p* = 0.013173 in the ICA group vs. the model group) in the serum of APP/PS1 mice (Fig. 4G, H). Altogether, these findings suggest that ICA modulates the gut metabolism and the consequent synthesis of metabolites, particularly sphingolipids and glycerophospholipids, which impact the host through circulation.

### Interaction between the gut microbiota and metabolites

**Fig. 5:**
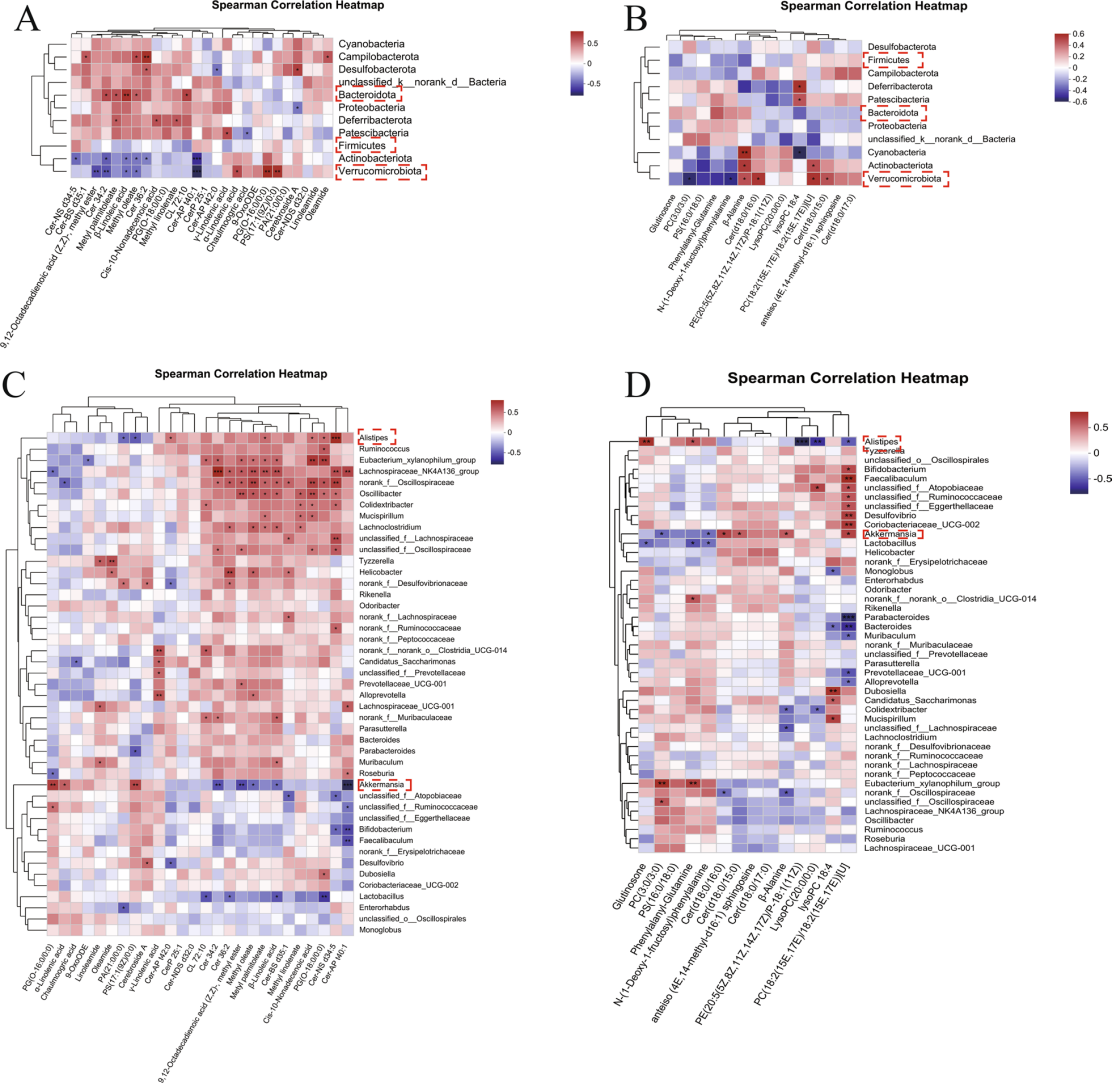
**A**, **C** Spearman correlation heatmaps of the gut microbiota and differential fecal metabolites, at the phylum **A** and genus **B** levels. **B**, **D** Spearman correlation heatmaps of the gut microbiota and differential serum metabolites, at the phylum **C** and genus **D** levels

Spearman analysis was performed between the gut microbiota and fecal/serum metabolites to further examine the anti-AD effects of ICA on the gut microbiota and metabolism. We found that the F/B ratio was significantly reduced in the ICA group compared to the model group and that the abundances of *Firmicutes* and *Bacteroidetes* were significantly correlated with sphingolipid metabolism (Cer(d18:0/15:0) in the serum and CerP 25:1 in the feces of the ICA group (Fig. 5A, B). Additionally, at the genus level, a few genera, including *Rikenella, Odoribacter, norank_f_Peptococcaceae, Parasutterella,* and *Enterorhabdus*, were not significantly correlated with any metabolites, while *Akkermansia* and *Alistipes* were significantly correlated with at least five metabolites in both the serum and fecal samples. Moreover, there was an obvious clustering phenomenon among the bacteria and metabolites. In the fecal samples (Fig. 5D), sphingolipids CerP 25:1, Cer-NDS d32:0, Cer-NS d34:5, and Cer 34:2 were negatively correlated with *Akkermansia* and positively correlated with *Alistipes*. In contrast, α-Linolenic and γ-Linolenic acids were positively correlated with *Akkermansia* and negatively correlated with *Alistipes*. Meanwhile in the serum samples (Fig. 5C), *Akkermansia*, in contrast with *Alistipes* and *Mucispirillum*, was positively correlated with sphingolipids Cer(d18:0/17:0) and Cer(d18:0/15:0) and negatively correlated with glycerophospholipids PC (3:0/3:0) and PS (16:0/18:0). Interestingly, *Verrucomicrobiota*, the phylum of *Akkermansia*, showed the same correlation with *Akkermansia*. These findings revealed that the reduced abundance of *Akkermansia* and increased abundance of *Alistipes* were associated with the increased levels of ceramides in the APP/PS1 mice. Moreover, the ceramide levels in APP/PS1 mice decreased when the abundances of *Akkermansia* and *Alistipes* were altered by ICA intervention. Altogether, these results indicate that the therapeutic effect of ICA may be strongly associated with sphingolipid metabolism and that *Akkermansia* and *Alistipes* may play a crucial role in this process, thus indicating that ICA mediates its anti-AD effect via the microbiome–metabolites–brain axis.

### Prediction of potential drug–target pathways associated with the anti-AD activity of ICA

**Fig. 6:**
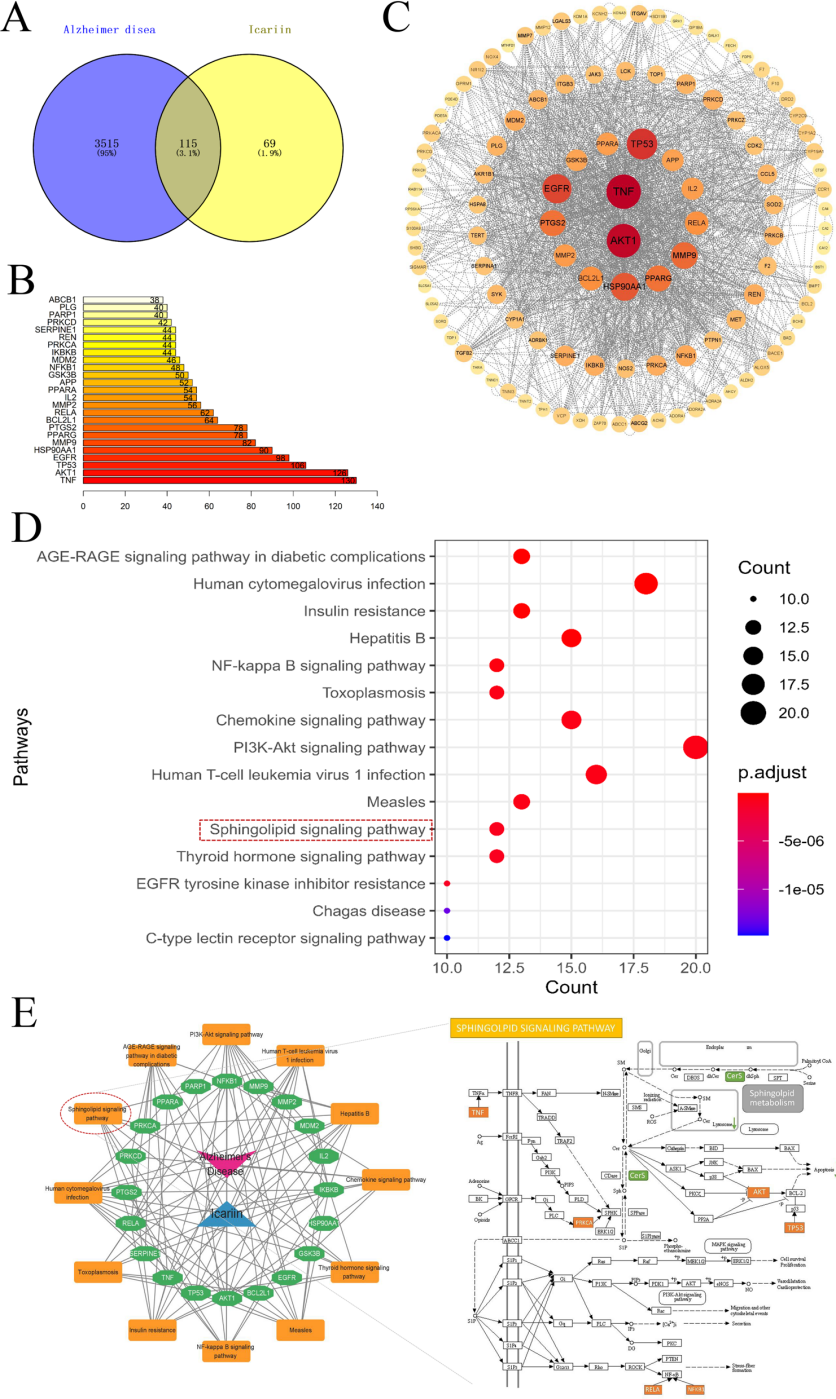
NP analysis for ICA in AD treatment. **A** Venn diagram of the ICA and AD target genes. **B** Analysis of the ICA and AD hub genes. **C** PPI network for ICA in AD treatment. The nodes represent functional proteins, the edges represent the interactions between the proteins, and the size and hue of the circles indicate the significance of the proteins. **D** KEGG enrichment analysis of the potential AD-associated ICA targets. The size of the dots represents the number of genes engaged in the pathway and the color represents the adjusted p-value. **E** Network of interactions between AD, icariin, targets, and pathways, in which red represents AD, blue represents icariin, green represents putative protein targets, and yellow represents the pathway. NP, network pharmacology; ICA, icariin; AD, Alzheimer's disease; PPI, protein–protein interaction; KEGG, Kyoto Encyclopedia of Genes and Genomes

NP analysis was performed to determine the anti-AD mechanism of ICA. Firstly, 184 drug targets were identified through PubChem, Pharm Mapper, and Swiss Target Prediction databases. Additionally, 3630 AD-related genes were obtained from the OMIM and GeneCards databases, among which 115 prospective ICA targets were selected for further analysis (Fig. 6A, Additional file 1: Table S1). As the proteins engaged in biochemical processes generate supramolecular compounds to mediate biological activities, it is essential to investigate the association of ICA with various proteins to characterize its pharmacological effects. Therefore, the candidate AD-related ICA targets were fed into the STRING database to determine the associated PPI network. The cut-off value was set to 0.9 and the unconnected proteins were excluded from the network. A PPI network was constructed with 111 nodes (representing functional proteins) and 3,100 edges (representing the interactions between functional proteins and other proteins) using Cytoscape (Fig. 6C). The top 25 hub genes in the PPI network, including TNF, AKT1, TP53, EGFR, and NFKB1, were determined based on the node degree (Fig. 6B) and these hub genes may be the crucial target genes of ICA in AD treatment.

KEGG enrichment analysis (*p* < 0.05) revealed that a total of 119 KEGG pathways, including the PI3K-Akt signaling pathway, sphingolipid signaling pathway, and NF-κB signaling pathway, were significantly enriched by the 115 target genes (Additional file 1: Table S2). Fig. 6D depicts the bubble plot of the 15 most important KEGG pathways associated with the target genes, which was used to construct a drug–target–pathway network. Notably, we found that ICA had a regulatory effect on the sphingolipid signaling pathway via the PRKCA/TNF/TP53/AKT1/RELA/NFKB1 axis (Fig. 6E). These predicted genes were selected for molecular docking analysis with ICA.

Molecular docking analysis demonstrated that ICA may readily access and bind to the active pocket of PRKCA and five additional proteins (Additional file 1: Figure S4), which combined with the metabolomics results, indicates that ICA could affect the sphingolipid signaling pathway via the PRKCA/TNF/TP53/AKT1/RELA/NFKB1 axis.

## Discussion

ICA, an active ingredient extracted from *Epimedium* species, has been reported to mediate its anti-AD effects by inhibiting phosphodiesterase-5 activity and decreasing external Aβ plaques and intracellular NFTs [4, 9]. Therefore, ICA might serve as a promising compound for AD treatment. Consistent with the previous studies, our study revealed that ICA reversed the cognitive impairment and histopathological alterations in the APP/PS1 mice [22, 23]. Furthermore, we found that the anti-AD function of ICA is associated with the restoration of gut microbiota dysbiosis and metabolite profile and that *Akkermansia* and *Alistipes* significantly affect sphingolipid metabolism. Our findings demonstrate the significance of the microbiota–metabolite–brain axis in the anti-AD effect of ICA, suggesting that it may serve as a promising therapeutic target for ICA-mediated AD treatment.

The gut is connected with the brain via the gut-nervous system and neuroinflammation [24]. Changes in the abundance of specific species of intestinal flora have been linked to AD progression [25]. For instance, Zhang et al. found that the abundance of *Ruminiclostridium_5*, which is related to systemic inflammation, was markedly elevated in the intestinal flora of the AD mice and that the transplantation of gut microbiome from the control mice alleviated inflammatory responses in the AD mice [26]. Furthermore, increasing evidence highlights the close association between ICA and the gut microbiota [9, 27].

Our work reveals that ICA has a significant influence on gut microbiome composition. For instance, at the phylum level, ICA restored gut dysbiosis by downregulating the F/B ratio, consistent with previous research [28]. Additionally, at the genus level, the abundances of *Alistipes* (associated with a higher-fat diet [20]) and *Mucispirillum* (associated with pro-inflammatory effects [21]) increased in the AD mice; however, they decreased after ICA treatment. In contrast, the abundance of *Akkermansia* (associated with anti-inflammatory effects and metabolic status [19]) was significantly elevated after ICA treatment. These results suggest that ICA may play a pharmacological role in AD by regulating the composition and function of metabolism- and inflammation-related flora.

The gut microbiome significantly influences the host's metabolism. For instance, *Akkermansia* regulates the gut–brain axis by maintaining the intestinal mucosal barrier and regulating lipid metabolism [19, 29]. Therefore, ICA-induced changes in the gut microbiota may further cause metabolic changes in the gut. Consistently, functional analysis of the intestinal microbiota and metabolomic profiling of the fecal samples demonstrated significant differences in the metabolic profiles, especially of sphingolipids, of the intestinal flora between the model and ICA groups. Upregulation of ceramides, the primary metabolites and second messengers of sphingolipids, can increase Aβ levels by regulating β-secretase [30] and γ-secretase [31] activities. Activation of sphingomyelinase, which catalyzes the breakdown of sphingomyelin to ceramides, triggers the formation of oligomers and fibrous Aβ, which further stimulate the increase in ceramide levels, constituting a positive feedback cycle [32]. Furthermore, several studies demonstrated the role of ceramides in neuronal death [33]. In our study, ceramide levels were decreased after ICA treatment, suggesting that ICA had positive effects on the composition and metabolic profile of the gut microbiome. In addition, serum metabolomics further proved that ICA could, at least partially, regulate lipid metabolism in AD mice.

Previous studies revealed that *Bacteroidetes* is a dominant phylum of the gut microbiome and that it is associated with sphingolipid production [34], suggesting that ICA may interfere with sphingolipid metabolism by regulating *Bacteroides*. Consistently, in our study, correlation analysis showed that the abundance of *Bacteroidetes* was significantly correlated with sphingolipid metabolism. Furthermore, *Akkermansia* and *Alistipes* were correlated with at least 5 metabolites, in both the serum and fecal samples and may be the primary gut microbial species to interact with the host’s metabolites. *Alistipes*, a comparatively novel sub-branch of the *Bacteroidetes* phylum, is often linked with persistent intestinal inflammation and sphingolipid production [34–36], consistent with our results. Therefore, an imbalance in Alistipes abundance is often associated with the onset of intestinal mucosal damage and lipid metabolism disorders [37]. In contrast, *Akkermansia* is associated with anti-inflammatory function in the intestines, reduction in obesity, and lipid metabolism regulation [38]. *Akkermansia* has been reported to promote Aβ40-42 reduction and improve intestinal barrier dysfunction and dyslipidemia in APP/PS1 mice [39]. In the present study, ICA therapy lowered ceramide levels in APP/PS1 mice, which was associated with an increase in *Akkermansia* abundance and a decrease in *Alistipes* abundance. Collectively, our findings underscored the significance of the microbiome–metabolite–brain axis in mediating the anti-AD effects of ICA.

Finally, NP was used to investigate the association between ICA and AD treatment based on system networks. The results revealed that the therapeutic effect of ICA on AD may be mediated via a variety of signaling pathways, including the PI3K-Akt signaling pathway, sphingolipid signaling pathway, and NF-κB signaling pathway, consistent with previous studies [40]. ICA has been reported to have a therapeutic effect on ovarian cancer by regulating the PI3K-Akt signaling pathway and NF-κB signaling pathway [41]. Consistent with the metabolomics results, ICA was hypothesized to affect the sphingolipid signaling pathway via the PRKCA/TNF/TP53/AKT1/RELA/NFKB1 axis (Fig. 6F). Sphingomyelin and its metabolites serve as second messengers in several cellular signaling pathways. Specifically, ceramides, a novel class of potent bioactive compounds, can stimulate several signaling pathways, including the PI3K/AKT and MAPK/ERK pathways, thus regulating different processes associated with neuronal survival and death [42]. Particularly, TNF-α has been considered an immune-mediated factor of the sphingolipid signaling pathway in AD [43] and has been associated with synaptic dysfunction in AD-related cognitive decline [44]. Additionally, phosphorylation of PRKCA, a member of the protein kinase C family, can stimulate SPHK1 and SPHK2 production to trigger ceramide-induced apoptosis [45]. RELA and NFKB1 encode a subunit of NF-κB that is involved in the stimulation of the downstream NF-κB signaling pathway [46]. Furthermore, AKT1 phosphorylation has been reported to induce the production of ceramide synthase 6, which catalyzes the formation of ceramides from sphingoid bases and acyl-CoA substrates through activation of the recombinant ceramide synthase 6 promoter [47]. p53, encoded by *TP53*, plays a crucial role in sphingolipid-induced cell apoptosis [48]. Molecular docking analysis, in our study, confirmed the affinity of these predicted proteins with ICA, suggesting that these proteins may serve as potential targets for ICA in AD treatment.

Some of the limitations of this study need to be acknowledged. Firstly, we used a single analytical method to detect the fecal and serum metabolites, which may limit the scope of our study; therefore, multiple analytical methods should be used in future studies to identify more metabolic pathways associated with the ICA-mediated treatment of AD. Secondly, we only explored the metabolomic changes in the fecal and serum samples before and after cognitive impairment in APP/PS1 mice; however, combined multi-site and multi-omics studies can more accurately determine the regulatory networks associated with the development of cognitive decline. Lastly, we used a single animal model and single drug dose in this study, and further studies should be conducted using different animal models, such as 5XFAD mice and 3xTg-AD mice, and under different drug doses, to further validate our results.

## Conclusions

This is the first study to explore the underlying mechanisms associated with the anti-AD function of ICA by integrated 16 s rRNA sequencing, multi-metabolomics, and NP analyses. Our results revealed that ICA can effectively improve AD-induced cognitive impairment and pathologic changes. In addition, ICA ameliorated AD-induced gut microbiome dysbiosis and metabolic disorder in APP/PS1 mice. Interestingly, we found an association between gut microbiota (*Alistipes* and *Akkermansia*) and sphingolipid metabolism, which might be key factors contributing to the therapeutic effect of ICA on AD. Furthermore, NP analysis revealed the potential mechanisms underlying the ICA-mediated treatment of AD that might affect the sphingolipid signaling pathway via the PRKCA/TNF/TP53/AKT1/RELA/NFKB1 axis. Altogether, our results indicate that ICA may serve as a potential therapeutic compound for preventing AD development via the microbiome–metabolite–brain axis. However, further studies, specifically multi-omics, spatial transcriptomics, and spatial metabolomics analyses are required to further explore the exact mechanism by which ICA mediates its therapeutic effect on AD.

## Supplementary Information

**Additional file 1: Figure S1.** Alpha diversity of each group. **Figure S2.** Fecal metabolomics supplement results. **Figure S3.** Serum metabolomics supplement results. **Figure S4.** Molecular docking: The pink molecule is icariin, and the blue structure is the binding site between icariin and protein macromolecules. **Table S1.** The information of the 115 intersecting target genes. **Table S2.** The information of KEGG pathway related target genes (Top 20).

## Data Availability

The dataset supporting the conclusions of this article is available in the National Center for Biotechnology Information Sequence Read Archive under BioProject ID: PRJNA918352.

## Abbreviations

16S rRNA: 16S ribosomal RNA
AD: Alzheimer's disease
ANOVA: Analysis of variance
COX: Cyclooxygenase
CYP: Cytochrome P
FC: Fold change
GABA: Gamma-amino butyric acid
GO: Gene ontology
GSK: Glycogen synthase kinase
HE: Hematoxylin–eosin
ICA: Icariin
KEGG: Kyoto encyclopedia of genes and genomes
LDA: Linear discriminant analysis
LEfSe: Linear discriminant analysis effect size
MWM: Morris water maze
NFT: Neurofibrillary tangle
NLRP6: Nucleotide-binding oligomerization domain: leucine-rich repeat and pyrin domain-containing 6
OPLS-DA: Orthogonal partial least-squares discriminant analysis
PCR: Polymerase chain reaction
PLS-DA: Partial least squares discrimination analysis
SIRT1: Silent mating type information regulation 2 homolog- 1
TNF: Tumor necrosis factor
UHPLC/TOF–MS: Ultrahigh-performance liquid chromatography/time-of-flight mass spectrometry
VIP: Variable importance in the projection
