# Supplementary material for: Anti-Alzheimers molecular mechanism of icariin: insights from gut microbiota, metabolomics, and network pharmacology
Source: J Transl Med. 2023 Apr 24;21:277. doi: 10.1186/s12967-023-04137-z (PMC10124026; doi:10.1186/s12967-023-04137-z)
Supplement: Supplementary file 1 — Additional file 1: Figure S1. Alpha diversity of each group. Figure S2. Fecal metabolomics supplement results. Figure S3. Serum metabolomics supplement results. Figure S4. Molecular docking: The pink molecule is icariin, and the blue structure is the binding site between icariin and protein macromolecules. Table S1. The information of the 115 intersecting target genes. Table S2. The information of KEGG pathway related target genes (Top 20). [file 12967_2023_4137_MOESM1_ESM.docx]

## Additional material

## Detailed Methods

### Preparation of herbal extracts

ICA powder (98% purity) was purchased from (Nanjing Spring & Autumn Biological Engineering Co., Ltd., CH33H40015). ICA was dissolved in 2% dimethyl sulfoxide (DMSO) and 10% polyethylene glycol 400.

### Animal modeling, grouping, and intervention

This study was carried out following the recommendations in the Guide for the Care and Use of Laboratory Animals of the National Institute of Health. All procedures that involved animals were approved by the Animal Ethics Committee of the Second Xiangya Hospital, Central South University (2020038). All procedures were conducted under pentobarbital sodium anesthesia, and discomfort was kept to a minimum. 250–300 g, 6-month-old C57BL6/J and APP/PS1 mice were obtained from Spife (Beijing) Biotechnology Co., Ltd. Every mouse was kept in a regulated habitat (50±10% relative humidity, 12/12 h light/dark cycle, 22±2°C) with free access to standard food and water. APP/PS1 mice were randomly allocated into two groups: model group (n=10) and ICA group (n=12), whereas C57BL6/J mice were assigned to the control group (n=10). The ICA group was administered ICA at a dosage of 100 g/(kg*d) for 100 days, whilst the other two groups received the same quantity of sterile saline solution

### Morris water-maze (MWM) test

The Morris water maze test, comprising a spatial acquisition test and spatial probe test, was employed to evaluate the spatial learning and memory abilities as previously described, with modifications. In brief, the Morris water maze apparatus consists of four equally spaced quadrants with different figures marked on the wall above each quadrant. A transparent platform (diameter: 4 cm; height: 58 cm) was placed in the center of one quadrant, and the water (depth: 60 cm) was maintained at a temperature between 22°C and 25°C. The spatial acquisition test was performed between days 96 and 100 after Aβ1-42 infusion to evaluate the spatial learning ability of the animals through a 5-day memory acquisition experiment. A subsequent spatial probe experiment was performed on day 99 to determine the spatial memory retention ability of rats. We applied behavioral video tracking systems (XR-XM101; Shanghai Softmaze Information Technology Co. Ltd., Shanghai, China) for tracking and analysis of experimental parameters. The investigator conducting the Morris water maze test was blinded to the grouping of the rats.

### Hematoxylin and eosin (HE) staining

The morphological changes of the CA1 area of the mouse hippocampus were observed by pathological examination. At the end of the MWM experiment, the rats were anesthetized and perfused transcardially with 300 mL of stroke-physiological saline solution to remove blood and their brain tissue was removed and quickly placed on the ice tray along the sagittal suture. The brain tissues were fixed in 4% formalin solution at 4°C for 8 h, taken out in a 70% ethanol solution for 5 min, then placed in 80%, 90%, 95%, and absolute ethanol for gradient dehydration for 4 h each time, respectively; finally, tissues were immersed in xylene for 30 min, and then embedded in paraffin. Continuous coronal sections at the optic chiasma area (including the hippocampus) were taken, and the slice thickness was 3 μm. Each specimen was taken for 10 consecutive slices for the HE tests.

### 4. 16S rRNA Gene Sequencing analysis

#### 4.1 Collection of intestinal contents

Fecal samples were collected at the age of 100 days, and at least 5 fecal pellets were obtained directly from the anus of each mouse, deposited into a sterile conical tube, and immediately frozen at −80°C until further analysis.

#### 4.2. Gut data acquisition and processing

DNA was extracted with the use of a QIAamp PowerFecal DNA kit (QIAGEN 12830), and the extracted genomic DNA was examined by electrophoresis on a 1% agarose gel. Subsequently, specific primers with barcode were synthesized according to the indicated sequencing region, and PCR was performed using TransGen AP221-02: TransStart Fastpfu DNA Polymerase in triplicate for each sample, PCR products from the same sample were pooled and detected by electrophoresis on a 2% agarose gel, and PCR products were recovered by cutting the gel with the AxyPrepDNA gel Recovery kit (AXYGEN). Sequencing was performed using the Illumina system (Illumina MiSeq, USA). The data were analyzed on the online platform of Majorbio Cloud Platform (www.majorbio.com). Chao, Shannon indices and analysis of bacterial abundant were determined using QIIME. And differences between two groups were visualized by PLS-DA plots. Statistically significant differences in the relative abundances of genera among groups were determined using a linear discriminant analysis (LDA) effect size (LEfSe) algorithm. LDA values > 2.5 with a P-value < 0.05 were considered significantly enriched. PICRUSt was used to predicte functional orthologs to identify functional changes among groups. The data have been deposited in the National Center for Biotechnology Information (NCBI) Sequence Read Archive (SRA) under BioProject ID: PRJNA918352.

### 5. LC-MS based metabolomics

#### 5.1 Feaces collection and preparation

After 100 days of administration, stool samples were taken from all mice in collection tubes containing a buffer that prevented the growth of microorganisms and stabilized DNA and stored at −80C°. When metabolomics was required, about 60mg of the frozen intestinal contents were taken out and placed into 1.5mLEP tube, 20uL of internal standard (L-2-chlorophanine, 0.3mg/mL, methanol as solvent) was added, and 600μL methanol-water (V: V=4:1) was added. Add two small steel balls, place them in the -20℃ refrigerator for 5min, and then grind them in the grinder (60Hz, 2min). Ultrasonic extraction was carried out in an ice bath for 10min and left for 30min at -20℃. Then centrifuge for 10min(13000rpm,4℃), take 200uL supernatant and put it into LC-MS sample vial. Redissolved with 300uL methanol-water (V:V=1:4), rotated for 30s, ultrasonic for 3min, and stood at -20℃ for 2 hours. Finally, after centrifugation for 10min(13000rpm, 4℃), 150uL of the supernatant was put into an LC-MS sample vial with a leg liner tube for analysis. Quality control sample (QC) is prepared by mixing the extract of all samples in equal volume, and the volume of QC is the same as that of the sample.

#### 5.2 Serum collection and preparation

And after the Morris water maze test, the rats were sacrificed under anesthesia and blood was collected through the abdominal aorta. For metabolomics analysis, the blood samples stored at -80℃ were taken out, thawed at room temperature, 100uL of samples were removed, 20μL of internal standard (L-2-chlorophanine, 0.3mg/mL, methanol as solvent) was added, and the vortex was oscillated for 10s. Then 300uL of protein precipitator methanol: acetonitrile (V: V=2:1) was added, and the vortex was oscillated for 1min. Then it was extracted by ultrasound in an ice bath for 10min and left for 30min at -20℃. After centrifugation for 10min(13000rpm, 4℃), 300uL of supernatant was put into LC-MS sample vial and dried. Redissolved with 300uL methanol-water (V:V=1:4), rotated for 30s, and stood at -20℃ for 2 hours after 3min of ultrasound; . After centrifugation for 10min(13000rpm, 4℃), 150uL of the supernatant was put into LC-MS sample vial with a leg liner tube for analysis. Quality control sample (QC) is prepared by mixing the extract of all samples in equal volume, and the volume of QC is the same as that of the sample.

#### 5.3 LC-MS data acquisition

UHPLC/TOF-MS analysis was performed on UltiMate 3000 Ultra High performance Liquid Chromatograph (Thermo Fisher USA) and Thermo Q-Exactive Orbitrap Mass Spectrometer (Thermo Fisher). Chromatographic separations were performed on Waters ACQUITY UPLC HSS T3（100 mm×2.1 mm, 1.8 um. The mobile phase consisted of solvent A (0.1% formic acid in water) and B (methanol). The solvent gradient program was as follows: 0-3min, 95% (A): 5% (B) - 70% (A): 30% (B); 3-5min, 70% (A): 30% (B) - 40% (A): 60% (B); 5-7min, 40% (A): 60% (B) - 20% (A): 80% (B); 7-12min, 20% (A): 80% (B) - 0% (A): 100% (B); 12-16.5min, 0% (A): 100% (B) - 95% (A): 5% (B). The column temperature was 45°C. The sample injection volume was 10μL and the flow rate was 0.35 mL/min. All samples were stored at 4°C during analysis. The source temperature was 320°C and the declustering potential was 80 V. For mass spectrometry analysis, HESI was used as the ion source, and positive and negative ion scanning mode was used for sample quality spectrum signal acquisition. Pre-processing and analysis of the UPLC-MS data were performed as described previously.

#### 5.4 Data processing

All raw data were then loaded into Progenesis QI software (Waters, Milford, MA, USA) and SIMCA-P14.0 software (Umetrics AB, Umea, Vasterbotten, Sweden) for further analysis. SIMCA-P 14.1(Umetrics, Sweden) was carried out for the partial least-squares discriminant analysis (PLS-DA) and orthogonal partial least-squares discriminant analysis (OPLS-DA). Variable importance in the projection (VIP) > 1 and P < 0.05 were selected as differential metabolites. For advanced data analysis, heatmap analysis and KEGG enrichment analysis were performed.

#### 5.5 Metabolic pathway analysis

Metabolic pathway analysis of differentially expressed metabolites detected through LC-MS was performed using MetaboAnalyst 5.0 (www.metaboanalyst.ca) to obtain insight into the biological mechanisms related to the treatment of ICA. MetaboAnalyst 5.0 is a free web-based quantitative metabolome data analysis platform with eight modules: statistical analysis, enrichment analysis, pathway analysis, power analysis, integrated pathway analysis, design, biomarker analysis, and other utilities. Metabolite data were imported into MetaboAnalyst 5.0 to investigate the neuroprotective mechanisms of ICA treatment on AD. The impact value threshold calculated from pathway topology analysis was set to 0.10, and a raw p value < 0.05 was regarded as significant.

### 6. Gut microbiota - metabolite correlation analysis

The top 20 differential metabolites between ICA and Model judged by Fold change (FC) were used for correlation analysis, with the top 50 species in the abundance of intestinal flora. Spearman correlation analysis was then performed and heat maps were made.

### 7. Network pharmacology

#### 7.1 Screening of drug targets and disease targets

The pharmacophore targets were predicted by PubChem (https://pubchem.ncbi.nlm.nih.gov/), Pharm Mapper (http://www.lilab-ecust.cn/pharmmapper/) and Swiss Target Prediction (http://www.swisstargetprediction.ch/). The 2D and 3D structures of ICA were obtained by PubChem, and the pictures were saved in an SDF format. Then, the 3D SDF format file was uploaded to the Pharm Mapper server. Next, the first 300 target proteins were obtained by the “reverse pharmacophore matching method”, and targets were acquired by taking the value of Z score >0.8. Simultaneously, Swiss Target Prediction was used to predict the related target proteins. Icariin’s SMILES were obtained from PubChem:

C[C@H]1[C@@H]([C@H]([C@H]([C@@H](O1)OC2=C(OC3=C(C2=O)C(=CC(=C3CC––C(C)C)O[C@H]4[C@@H]([C@H]([C@@H]([C@H](O4)CO)O)O)O)O)C5=CC––C(C––C5)OC)O)O)O, then SMILES was entered into the Swiss Target Prediction database, and “Homo sapiens” was selected as the species to obtain 100 targets. All the retrieved targets were converted to their official symbols using the UniProt Knowledgebase search function (http://www.uniprot.org/) in the Protein Database (UniProt). After eliminating the repetition and coaction data, we obtained 184 target genes.

Gene Cards (https://www.genecards.org/) and OMIM (https://omim.org/) were utilized to collect the AD-related disease genes and targets. By retrieving the keywords “Alzheimer's disease” in sequence, 3202 target genes were obtained from Gene Cards limiting relevance score≥5.2, and 554 disease targets from OMIM. After excluding the repeated data, 3630 disease targets were acquired. After executing the venny.R order, 115 common targets were extracted from the two documents.

#### 7.2. Enrichment analysis and network construction

Kyoto Encyclopedia of Genes and Genomes (KEGG) pathway enrichment analyses were further performed to probe the biological function and potential mechanisms of the detected targets. Meanwhile, the acquired target genes were submitted to the Search Tool for the Retrieval of Interacting Genes (http://string-db.org/) to obtain a protein–protein interaction network. Furthermore, a potential compounds–potential targets–potential pathways network of ICA for treating AD was constructed by utilizing the network visualization software Cytoscape (http://cytoscape.org/, ver. 3.7.1).

#### 7.3. Molecular docking

The most significant gene from two critical subnetworks was selected for subsequent molecular docking analysis. The receptor protein coded by the selected gene was searched in the Uniprot database (https://www.uniprot.org/). We downloaded 3D structure of the protein in RCSB PDB database (https://www.rcsb.org/). The 3D structure for the molecule ligands was downloaded from the PubChem database (https://pubchem.ncbi.nlm.nih.gov/). PyMOL 2.5.0 software was performed the dehydration of the receptor protein and Autodock software was used to carry out hydrogenation and charge calculation of proteins. Parameters of the receptor protein docking site were set to include the active pocket sites where small molecule ligands bind. Finally, PyMOL 2.5.0 was used to dock the receptor protein with the small molecule ligands of ICA.

**8. Statistical analysis**

SPSS 20.0 software was used to analyze the data presented as the mean±standard deviation (SD) (IBM, Armonk, NY, USA). The escape latency was analyzed by separate repeated measures of two-way analysis of variance (ANOVA). Other data were evaluated by one-way ANOVA. P < 0.05 was deemed statistically significant.

## Additional figures


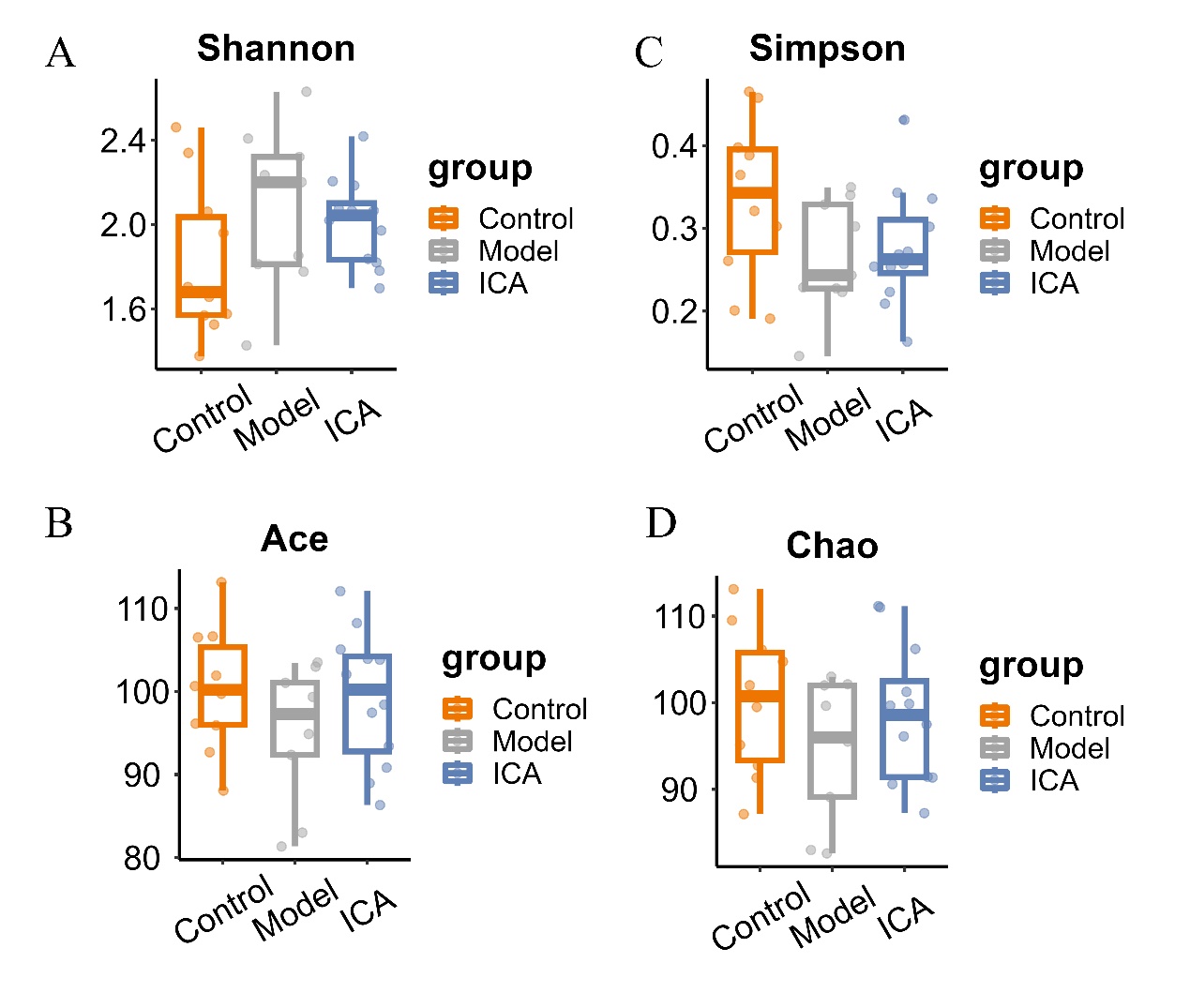


Figure S1 Alpha diversity of each group.


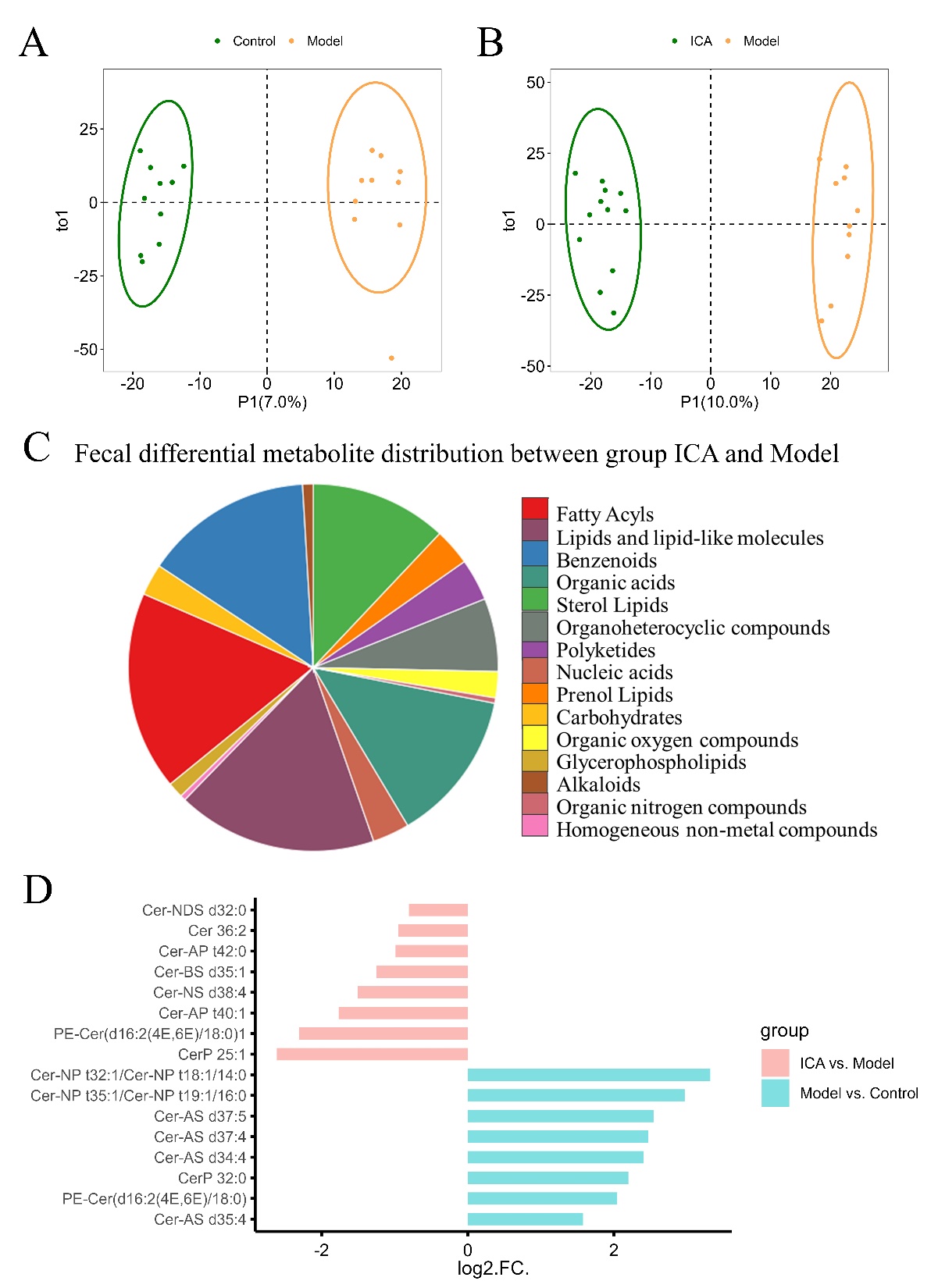


Figure S2 Fecal metabolomics supplement results. A) and B) are OPLS-DA analyses between the two groups. C) Differential metabolite distribution between the ICA group and the model group. D) Differential expression of ceramide metabolites between groups.


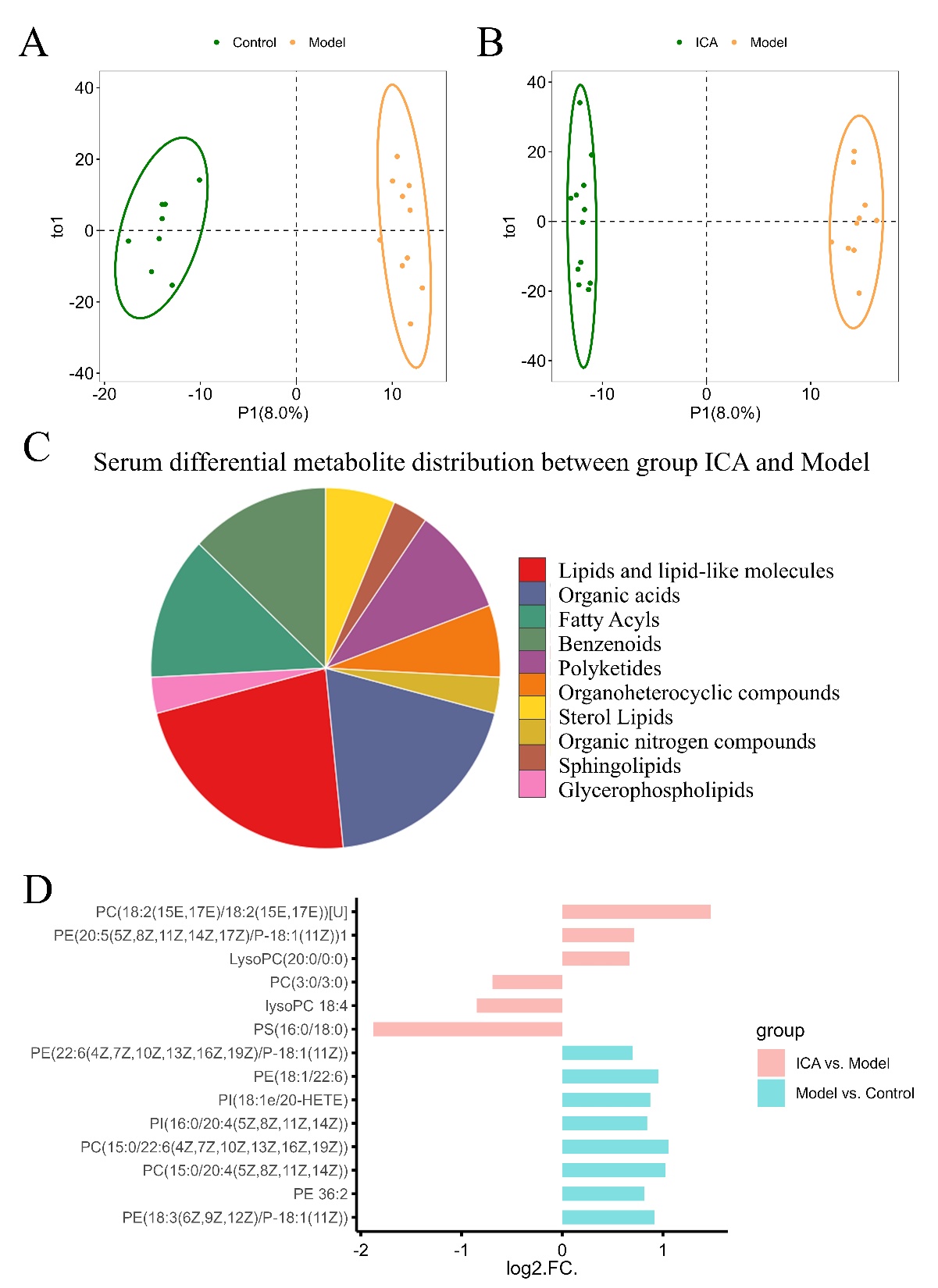


Figure S3 Serum metabolomics supplement results. A) and B) are OPLS-DA analyses between the two groups. C) Differential metabolite distribution between the ICA group and the model group. D) Differential expression of ceramide metabolites between groups.


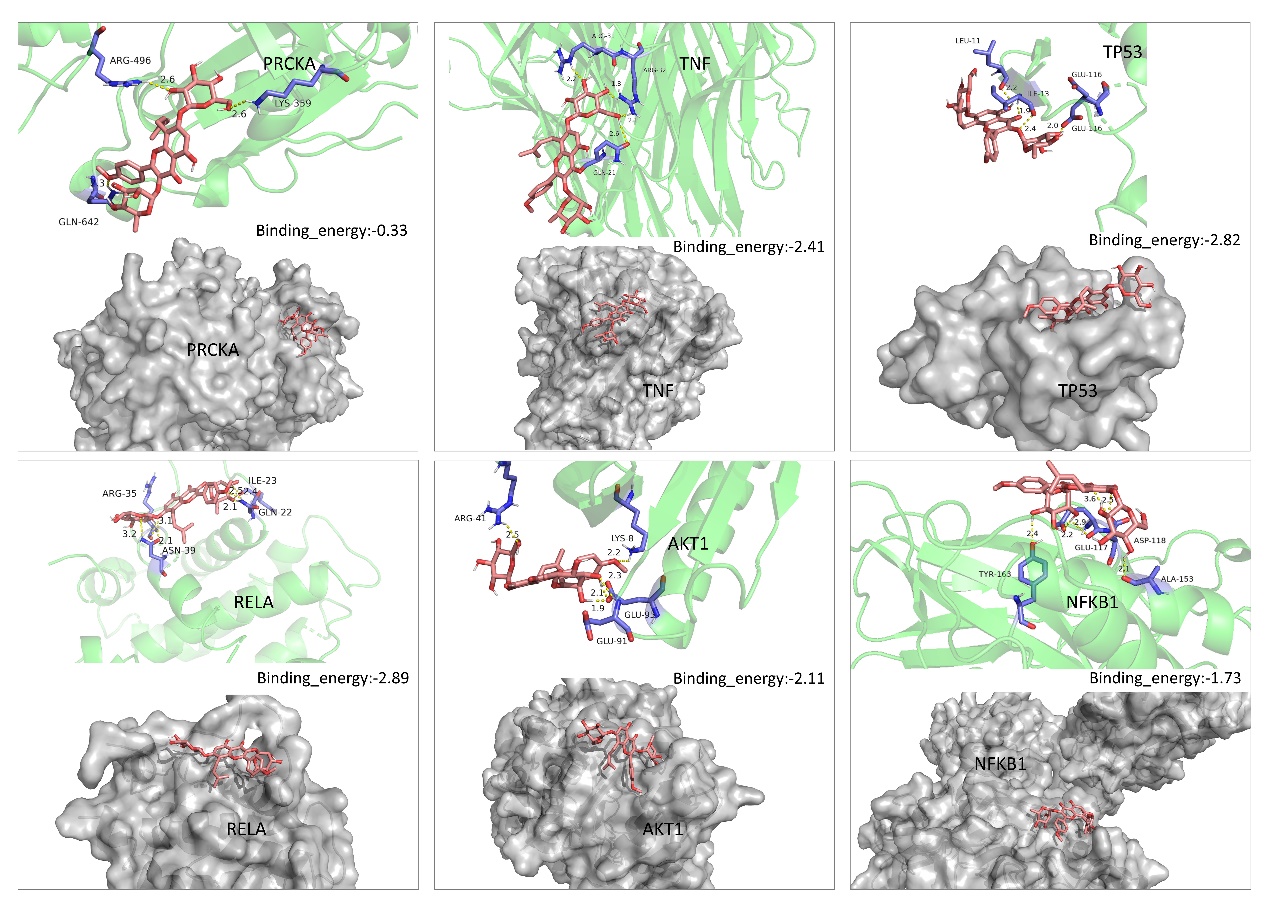


Figure S4 Molecular docking: The pink molecule is icariin, and the blue structure is the binding site between icariin and protein macromolecules. The connected hydrogen bonds are marked in yellow. The distance of each hydrogen bond is calculated respectively.

SUPPLEMENT TABLES

| NO. | Gene Name | Protein Name | NO. | Gene Name | Protein Name |
| --- | --- | --- | --- | --- | --- |
| 1 | PDE5A | cGMP-specific 3',5'-cyclic phosphodiesterase | 59 | EGFR | Epidermal growth factor receptor |
| 2 | AKR1B1 | Aldose reductase | 60 | PPARG | Peroxisome proliferator-activated receptor gamma |
| 3 | RPS6KA3 | Ribosomal protein S6 kinase alpha-3 | 61 | ADORA2A | Adenosine A2a receptor |
| 4 | NQO2 | Ribosyldihydronicotinamide dehydrogenase [quinone] | 62 | APP | Amyloid-beta A4 protein |
| 5 | CA12 | Carbonic anhydrase 12 | 63 | BACE1 | Beta-site app-cleaving enzyme 1 (memapsin 2) |
| 6 | CA4 | Carbonic anhydrase 4 | 64 | SLC5A1 | Sodium/glucose cotransporter 1 |
| 7 | NOX4 | NADPH oxidase 4 | 65 | RELA | Rela proto-oncogene, nf-kb subunit |
| 8 | PTGS2 | Prostaglandin G/H synthase 2 | 66 | SIGMAR1 | Sigma non-opioid intracellular receptor 1 |
| 9 | CA2 | Carbonic anhydrase 2 | 67 | DRD2 | D(2) dopamine receptor |
| 10 | ACHE | Acetylcholinesterase (cartwright blood group) | 68 | NFKB1 | Nuclear factor NF-kappa-B p105 subunit |
| 11 | ADRA2A | Alpha-2A adrenergic receptor | 69 | CYP1A1 | Cytochrome p450 family 1 subfamily a polypeptide 1 |
| 12 | PRKCD | Protein kinase C delta type | 70 | CYP1A2 | Cytochrome p450 family 1 subfamily a polypeptide 2 |
| 13 | PRKCA | Protein kinase C alpha type | 71 | OPRM1 | Mu-type opioid receptor |
| 14 | PRKCB | Protein kinase C beta type | 72 | SLC5A2 | Sodium/glucose cotransporter 2 |
| 15 | PRKCH | Protein kinase C eta type | 73 | ABCG2 | ATP-binding cassette sub-family G member 2 |
| 16 | TNF | Tumor necrosis factor | 74 | CDK2 | Cyclin-dependent kinase 2 |
| 17 | IL2 | Interleukin-2 | 75 | SHBG | Sex hormone-binding globulin |
| 18 | ADORA1 | Adenosine receptor A1 | 76 | PYGL | Glycogen phosphorylase, liver form |
| 19 | BCL2 | Apoptosis regulator Bcl-2 | 77 | PDE4D | cAMP-specific 3',5'-cyclic phosphodiesterase 4D |
| 20 | PRKCG | Protein kinase C gamma type | 78 | BMP7 | Bone morphogenetic protein 7 |
| 21 | XDH | Xanthine dehydrogenase/oxidase | 79 | HSPA8 | Heat shock cognate 71 kDa protein |
| 22 | TP53 | Cellular tumor antigen p53 | 80 | SORD | L-iditol 2-dehydrogenase |
| 23 | SERPINE1 | Plasminogen activator inhibitor 1 | 81 | REN | Renin |
| 24 | ALOX5 | Arachidonate 5-lipoxygenase | 82 | SOD2 | Superoxide dismutase [Mn], mitochondrial |
| 25 | PRKACA | cAMP-dependent protein kinase catalytic subunit alpha | 83 | MMP12 | Matrix metalloproteinase-12 (macrophage elastase) |
| 26 | VCP | Transitional endoplasmic reticulum ATPase | 84 | AHCY | Adenosylhomocysteinase |
| 27 | TNNC1 | Troponin C, slow skeletal and cardiac muscles | 85 | GALK1 | Galactokinase |
| 28 | TNNT2 | Troponin T, cardiac muscle | 86 | HSD11B1 | Corticosteroid 11-beta-dehydrogenase isozyme 1 |
| 29 | TNNI3 | Troponin I, cardiac muscle | 87 | PARP1 | Poly [ADP-ribose] polymerase 1 |
| 30 | LCK | Tyrosine-protein kinase Lck | 88 | GSK3B | Glycogen synthase kinase-3 beta |
| 31 | PRKCZ | Protein kinase C zeta type | 89 | MMP7 | Matrix metalloproteinase-7 (matrilysin, uterine) |
| 32 | F10 | Coagulation factor X | 90 | SERPINA1 | Alpha-1-antitrypsin |
| 33 | GRK2 | Beta-adrenergic receptor kinase 1 | 91 | MET | Hepatocyte growth factor receptor |
| 34 | GRK1 | Rhodopsin kinase | 92 | MDM2 | E3 ubiquitin-protein ligase Mdm2 |
| 35 | HSP90AA1 | Heat shock protein HSP 90-alpha | 93 | ABO | Abo, alpha 1-3-n-acetylgalactosaminyltransferase and alpha 1-3-galactosyltransferase |
| 36 | TERT | Telomerase reverse transcriptase | 94 | SYK | Spleen associated tyrosine kinase |
| 37 | F2 | Prothrombin | 95 | MTHFD1 | C-1-tetrahydrofolate synthase, cytoplasmic |
| 38 | F7 | Coagulation factor VII | 96 | CYP2C9 | Cytochrome p450 family 2 subfamily c polypeptide 9 |
| 39 | NOS2 | Nitric-oxide synthase, inducible | 97 | JAK3 | Tyrosine-protein kinase JAK3 |
| 40 | ITGAV | Integrin alpha-V | 98 | HEXB | Beta-hexosaminidase subunit beta |
| 41 | ITGB3 | Integrin beta-3 | 99 | PPARA | Peroxisome proliferator-activated receptor alpha |
| 42 | IKBKB | Inhibitor of nuclear factor kappa-B kinase subunit beta | 100 | LGALS3 | Galectin-3 |
| 43 | CYP19A1 | Aromatase | 101 | RAB11A | Ras-related protein Rab-11A |
| 44 | BAD | Bcl2-associated agonist of cell death | 102 | CCL5 | C-C motif chemokine 5 |
| 45 | BCL2L1 | Bcl-2-like protein 1 | 103 | BST1 | ADP-ribosyl cyclase/cyclic ADP-ribose hydrolase 2 |
| 46 | ALDH2 | Aldehyde dehydrogenase, mitochondrial | 104 | THRA | Thyroid hormone receptor alpha |
| 47 | TDP1 | Tyrosyl-DNA phosphodiesterase 1 | 105 | NR1I2 | Nuclear receptor subfamily 1 group I member 2 |
| 48 | KDM1A | [histone h3]-n6,n6-dimethyl-l-lysine4 fad-dependent demethylase | 106 | MMP2 | Matrix metalloproteinase-2 (gelatinase a) |
| 49 | AKT1 | RAC-alpha serine/threonine-protein kinase | 107 | FDPS | Farnesyl pyrophosphate synthase |
| 50 | KCNH2 | Potassium voltage-gated channel subfamily H member 2 | 108 | TPH1 | Tryptophan 5-monooxygenase |
| 51 | CCR1 | C-C chemokine receptor type 1 | 109 | ZAP70 | Tyrosine-protein kinase ZAP-70 |
| 52 | TOP1 | DNA topoisomerase 1 | 110 | S100A9 | Protein S100-A9 |
| 53 | PTPN1 | Tyrosine-protein phosphatase non-receptor type 1 | 111 | MMP9 | Matrix metalloproteinase-9 |
| 54 | ABCB1 | Multidrug resistance protein 1 | 112 | FECH | Ferrochelatase, mitochondrial |
| 55 | KCNA3 | Potassium voltage-gated channel subfamily A member 3 | 113 | GP1BA | Platelet glycoprotein Ib alpha chain |
| 56 | PLG | Plasminogen | 114 | CTSF | Cathepsin F |
| 57 | BCHE | Butyrylcholinesterase | 115 | TGFB2 | Transforming growth factor beta-2 |
| 58 | ABCC1 | Multidrug resistance-associated protein 1 |  |  |  |

Table S1 The information of the 115 intersecting target genes.

| ID | Description | pvalue | geneID |
| --- | --- | --- | --- |
| hsa04933 | AGE-RAGE signaling pathway in diabetic complications | 4.82E-10 | NOX4/PRKCD/PRKCA/PRKCB/TNF/BCL2/SERPINE1/PRKCZ/AKT1/RELA/NFKB1/MMP2/TGFB2 |
| hsa05163 | Human cytomegalovirus infection | 6.24E-10 | PTGS2/PRKCA/PRKCB/TNF/PRKCG/TP53/PRKACA/ITGAV/ITGB3/IKBKB/AKT1/CCR1/EGFR/RELA/NFKB1/GSK3B/MDM2/CCL5 |
| hsa04931 | Insulin resistance | 1.28E-09 | RPS6KA3/PRKCD/PRKCB/TNF/PRKCZ/IKBKB/AKT1/PTPN1/RELA/NFKB1/PYGL/GSK3B/PPARA |
| hsa05161 | Hepatitis B | 2.59E-09 | PRKCA/PRKCB/TNF/BCL2/PRKCG/TP53/IKBKB/BAD/AKT1/RELA/NFKB1/CDK2/JAK3/MMP9/TGFB2 |
| hsa04064 | NF-kappa B signaling pathway | 9.43E-09 | PTGS2/PRKCB/TNF/BCL2/LCK/IKBKB/BCL2L1/RELA/NFKB1/PARP1/SYK/ZAP70 |
| hsa05145 | Toxoplasmosis | 2.21E-08 | TNF/BCL2/ALOX5/NOS2/IKBKB/BAD/BCL2L1/AKT1/RELA/NFKB1/HSPA8/TGFB2 |
| hsa04062 | Chemokine signaling pathway | 2.67E-08 | PRKCD/PRKCB/PRKACA/PRKCZ/GRK2/GRK1/IKBKB/BAD/AKT1/CCR1/RELA/NFKB1/GSK3B/JAK3/CCL5 |
| hsa04151 | PI3K-Akt signaling pathway | 2.71E-08 | PRKCA/IL2/BCL2/TP53/HSP90AA1/ITGAV/ITGB3/IKBKB/BAD/BCL2L1/AKT1/EGFR/RELA/NFKB1/CDK2/GSK3B/MET/MDM2/SYK/JAK3 |
| hsa05166 | Human T-cell leukemia virus 1 infection | 2.77E-08 | TNF/IL2/TP53/PRKACA/LCK/TERT/IKBKB/BCL2L1/AKT1/RELA/NFKB1/CDK2/MMP7/JAK3/FDPS/TGFB2 |
| hsa05162 | Measles | 2.88E-08 | IL2/BCL2/TP53/IKBKB/BAD/BCL2L1/AKT1/RELA/NFKB1/CDK2/HSPA8/GSK3B/JAK3 |
| hsa04071 | Sphingolipid signaling pathway | 4.39E-08 | PRKCA/PRKCB/TNF/ADORA1/BCL2/PRKCG/TP53/PRKCZ/AKT1/ABCC1/RELA/NFKB1 |
| hsa04919 | Thyroid hormone signaling pathway | 5.30E-08 | PRKCA/PRKCB/PRKCG/TP53/PRKACA/ITGAV/ITGB3/BAD/AKT1/GSK3B/MDM2/THRA |
| hsa01521 | EGFR tyrosine kinase inhibitor resistance | 7.10E-08 | PRKCA/PRKCB/BCL2/PRKCG/BAD/BCL2L1/AKT1/EGFR/GSK3B/MET |
| hsa05142 | Chagas disease | 8.16E-07 | TNF/IL2/SERPINE1/NOS2/IKBKB/AKT1/RELA/NFKB1/CCL5/TGFB2 |
| hsa04625 | C-type lectin receptor signaling pathway | 9.79E-07 | PTGS2/PRKCD/TNF/IL2/IKBKB/AKT1/RELA/NFKB1/MDM2/SYK |
| hsa05165 | Human papillomavirus infection | 1.31E-06 | PTGS2/TNF/TP53/PRKACA/PRKCZ/TERT/ITGAV/ITGB3/IKBKB/BAD/AKT1/EGFR/RELA/NFKB1/CDK2/GSK3B/MDM2 |
| hsa04066 | HIF-1 signaling pathway | 1.51E-06 | PRKCA/PRKCB/BCL2/PRKCG/SERPINE1/NOS2/AKT1/EGFR/RELA/NFKB1 |
| hsa04210 | Apoptosis | 1.56E-06 | TNF/BCL2/TP53/IKBKB/BAD/BCL2L1/AKT1/RELA/NFKB1/PARP1/CTSF |
| hsa01523 | Antifolate resistance | 2.12E-06 | TNF/IKBKB/ABCC1/RELA/NFKB1/ABCG2 |
| hsa04722 | Neurotrophin signaling pathway | 3.38E-06 | RPS6KA3/PRKCD/BCL2/TP53/IKBKB/BAD/AKT1/RELA/NFKB1/GSK3B |

Table S2 The information of KEGG pathway related target genes (Top 20).
